# Postpartum haemorrhage (PPH) rates in randomized trials of PPH prophylactic interventions and the effect of underlying participant PPH risk: a meta-analysis

**DOI:** 10.1186/s12884-020-2719-3

**Published:** 2020-02-13

**Authors:** Lydia Hawker, Andrew Weeks

**Affiliations:** Sanyu Research Unit, Department of Women’s and Children’s Health, University of Liverpool, Liverpool, UK

**Keywords:** Postpartum haemorrhage, PPH risk, Population risk, PPH prophylaxis, PPH prevention

## Abstract

**Background:**

Postpartum haemorrhage (PPH) remains a leading cause of maternal mortality. Many trials assessing interventions to prevent PPH base their data on low risk women. It is important to consider the impact data collection methods may have on these results. This review aims to assess trials of PPH prophylaxis by grading trials according to the degree of risk status of the population enrolled in these trials and identify differences in the PPH rates of low risk and high risk populations.

**Methods:**

Systematic review and meta-analysis using a random-effects model. Trials were identified through CENTRAL. Trials were assessed for eligibility then graded according to antenatal risk factors and method of birth into five grades. The main outcomes were overall trial rate of minor PPH (blood loss ≥500 ml) and major PPH (> 1000 ml) and method of determining blood loss (estimated/measured).

**Results:**

There was no relationship between minor or major PPH rate and risk grade (Kruskal-Wallis: minor - T = 0.92, *p* = 0.82; major - T = 0.91, *p* = 0.92). There was no difference in minor or major PPH rates when comparing estimation or measurement methods (Mann-Whitney: minor - U = 67, *p* = 0.75; major - U = 35, *p* = 0.72). There was however a correlation between % operative births and minor PPH rate, but not major PPH (Spearman r = 0.32 v. Spearman r = 0.098).

**Conclusions:**

Using data from trials using low risk women to generalise best practice guidelines might not be appropriate for all births, particularly complex births. Although complex births contribute disproportionately to PPH rates, this review showed they are often underrepresented in trials. Despite this, there was no difference in reported PPH rates between studies conducted in high and low risk groups. Method of birth was shown to be an important risk factor for minor PPH and may be a better predictor of PPH than antenatal risk factors. Women with operative births are often excluded from trials meaning a lack of data supporting interventions in these women. More focus on complex births is needed to ensure the evidence base is relevant to the target population.

## Background

Postpartum haemorrhage (PPH) remains a leading obstetric emergency, causing 25% maternal deaths worldwide [1]. It is defined by the Royal College of Obstetricians and Gynaecologists (RCOG) as blood loss from the genital tract within the first 24 h after birth of at least 500 ml (minor) and at least 1000 ml (major) [2].

Causes of PPH are separated into the 4 ‘T’s – tone, thrombin, trauma and tissue [2]. Common causes include uterine atony (tone), preeclampsia (thrombin), perineal laceration (trauma) and retained placenta (tissue). The RCOG recognize several risk factors for PPH (Table 1) [2], however, this is not an exhaustive list and associations have been found with other risk factors including foetal death, maternal age and history of bleeding during pregnancy (antepartum haemorrhage).

**Table 1:** RCOG list of recognised risk factor for postpartum haemorrhage

Multiple pregnancy	Retained placenta
Previous postpartum haemorrhage	Perineal laceration
Pre-eclampsia	Episiotomy
Foetal macrosomia	General anaesthesia
Failure to progress (2nd stage)	Elective or emergency caesarean section
Prolonged third stage	
Placenta accrete	

NICE currently recommend advising active over physiological management due to its lowered risk of severe PPH (13/1000 vs 29/1000) [3]. Active management includes the use of uterotonic drugs and controlled cord traction. For women who progress to PPH, treatment involves further use of uterotonics, uterine massage, intravenous fluid, bladder emptying and controlled cord traction. Current evidence suggests the first line drug in this instance should be oxytocin, ergometrine or a combination of these [3].

Weeks and Nielson suggest that the evidence used to recommend treatment may not be relevant to many women who go on to suffer a PPH [4]. They suggest that the current evidence base is centred on uncomplicated births – with women at low risk of PPH – which only represents a small proportion of major PPH cases [5]. Those with more risk factors and complicated births may require a different approach in terms of treatment. Therefore, PPH research requires a global effort to refocus the efforts of PPH trials to access and improve the treatment for those in more at-risk groups.

Baseline variation in trial rates may be related to methods of blood loss measurement. Visual estimation is thought to be the preferred method as it is rapid and does not require practical equipment. However, comparison with objective methods has identified inaccuracies with visual estimation [6]. Hancock et al. performed a systematic review of blood loss measurement which found that estimation of blood loss tended towards overestimation of smaller volumes but increasingly underestimated larger volumes [6]. It may be necessary to standardise measurement of blood loss postpartum for what is a ‘crucial step’ to ensure rapid treatment and a reduction in maternal mortality.

The aim of this current review is to assess PPH rates in trials of prophylactic interventions for PPH, based on the PPH risk status of the trial populations. The rates between higher and lower risk trial populations will be compared. We will also consider the impact that blood loss assessment methods may have on these results.

## Methods

The protocol for this review was registered with PROSPERO (**CRD42018082322**).

### Trials and interventions

Randomised-controlled trials and cluster-randomised trials of intervention for the prevention of PPH were included in this study. Any intervention which acts to prevent PPH, administered in the third stage of labour, was included. The intervention could be pharmacological, examples include misoprostol, carbetocin, oxytocin, tranexamic acid and syntometrine, or non-pharmacological, including nipple stimulation, controlled cord traction and uterine massage. Interventions which related to the treatment or management of PPH were excluded as well as those interventions specifically related to the prevention, treatment and management of retained placenta.

### Identification of trials

The search was conducted through the Cochrane Central Register of Controlled Trials (CENTRAL) using the following search strategy; ‘randomised controlled trials’ and ‘third stage of labour or postpartum haemorrhage’ and ‘prophylactic or preventative or prophylaxis’. Published data from eligible trials was then included into further analysis. CENTRAL has a detailed search strategy which includes hand and electronic searches of PubMed, Embase, and ClinicalTrials.gov databases [7].

### Assessment and eligibility

The PRISMA (Preferred Reporting Items for Systematic Reviews and Meta-Analysis) guidelines were used as the basis for assessment. Trials were assessed for eligibility against five criteria:

1. Trials are appropriately randomised - randomised controlled trial (RCT) or cluster-randomised only
2. Trials report on the accepted definition of minor and major PPH (RCOG guidelines), or the equivalent value in peripartum fall in haemoglobin.
3. Trials identify their blood loss assessment method
4. Trials report the number of PPH cases in each arm
5. Trials report the baseline participant characteristics.

The definition used for PPH for this review is blood loss greater than or equal to 500 ml (minor) or greater than 1000 ml (major) [2]. An equivalent measurement for blood loss of 500 ml and 1000 ml is the drop of haemoglobin by 10% (10 g/l) or 20% (20 g/l) respectively. This assumes a rise in plasma volume during pregnancy giving a total blood volume at term of approximately 5000mls [8] and that a loss of 10% of this volume (5000mls) will also reduce the haemoglobin by this amount. Trials using this measurement and definition were also included.

### Risk of bias analysis

The risk of bias for included trials was assessed using the criteria outlined within the Cochrane Handbook for Systematic Reviews of Intervention [7]. If possible, risk of bias assessments from previously published reviews were used and have been appropriately identified in the assessment tables. Where no previous risk of bias assessment was found, the risk of bias was assessed independently by LH and AW.

Assessments have been made as low, unclear or high risk using the following domains;

1. **Random sequence generation (selection bias) –** randomisation methods were assessed to determine risk of bias.
2. **Allocation concealment (selection bias)** – allocation concealment methods and the risk of advanced allocation knowledge were assessed.
3. **Blinding of the participants and personnel (performance bias) –** methods to blind both participants and personnel to the allocated trial arm were assessed.
4. **Blinding of outcome assessment (detection bias) –** methods used to blind the outcome assessors to the trial arm and the objectivity of outcomes were assessed.
5. **Incomplete outcome data (attrition bias) –** missing outcome data and attrition rates were assessed.
6. **Selective reporting (reporting bias)** – where possible the published registration was assessed to determine selective reporting. Assessment was also made based on the expected reported outcomes for the trial.
7. **Other bias** – addressed on an individual trial basis where concerns over the risk of bias were identified.

### Data synthesis and outcomes

Eligible trials were graded according to the antenatal risk factors included in their study population and the method of delivery, using an original grading system developed by the authors based on risk factors in the RCOG guidelines [2] (Table 2). Method of delivery was assessed as a percentage of operative births (operative vaginal or caesarean section). In relation to antenatal risk factors, studies were classed as low risk or unselected. Low risk studies were those which defined themselves as low risk, stated that women with postpartum risk factors were purposely excluded or excluded at least 4 of the following; multiple pregnancy, previous PPH, preeclampsia, fibroids, polyhydramnios, intrauterine foetal demise, antepartum haemorrhage and placenta praevia/accreta (*for caesarean section only*).

**Table 2:** Grading system for trials of intervention for PPH prophylaxis, based on participant inclusion criteria

Grade	Definition
1	Only includes data from low risk women antenatally who have normal vaginal births
2	Includes data from women who are low risk antenatally and who have less than 10% operative births
3	Includes women who are low risk antenatally but who have 10% or more operative births
4	Includes data from unselected antenatal women where all births are included irrespective of rate of operative birth
5	Includes data from unselected antenatal women, all of whom have operative births.

The primary outcome was the average rate of PPH (minor and major) for each grade. As a secondary outcome, the method of calculating blood loss and determining PPH was also identified and analysed.

### Statistical analysis

For each grade and severity of PPH, a random effects pooled proportion (the number of women with PPH in relation to the whole trial population) with 95% confidence intervals (CI) was calculated using proportion meta-analysis. Statistical heterogeneity was assessed using the *I*^*2*^ statistic where > 30% was considered low, > 50% considered medium and > 75% considered high heterogeneity, a *p*-value < 0.1 was considered significant [7]. An overall pooled proportion across all grades for minor and major PPH was also generated and relationship between grade and pooled proportion was analysed using the Kruskal-Wallis test, where *p* < 0.05 was considered significant. To compare blood loss estimation and measurement, the Mann-Whitney U test was performed within each grade where appropriate, and for overall comparison of rates for minor and major PPH (α = 0.05). Post-hoc analysis using Spearman Rank Correlation was also carried out to identify any significant correlation between the percentage of operative births and proportion of PPH across all included studies. A change from the registered protocol was the use of StatsDirect3 to carry out statistical analysis.

## Results

### Search results

**Fig. 1:**
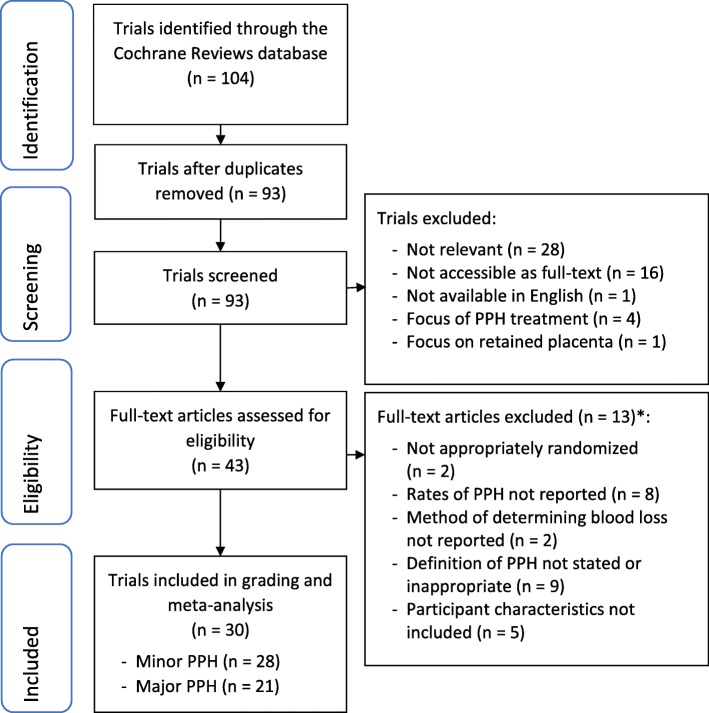
PRISMA diagram for the selection and eligibility assessment of trials focussing on prophylactic postpartum haemorrhage interventions. *Some full-text articles were excluded for not meeting more than one inclusion criteria. PPH – postpartum haemorrhage; PRISMA - Preferred Reporting Items for Systematic Reviews and Meta-Analysis

The search through the Cochrane Reviews database identified 93 trials which should be screened and assessed for eligibility (Fig. 1). Of these 93 trials, 30 were eligible for inclusion for grading and meta-analysis.

There were 28 trials which reported results for the minor PPH outcome and 21 trials for major PPH. Assessment of blood loss was measured or calculated using Hb only in 22 trials and was estimated only in seven trials. One trial reported estimated blood loss ≥500 ml (minor PPH), and a measured Hb of 2 g/dL (major PPH) [9].

Of the excluded trials, 50 were excluded during screening and a further 13 were excluded as they did not meet the review eligibility criteria.

### Risk of bias of included trials

**Fig. 2:**
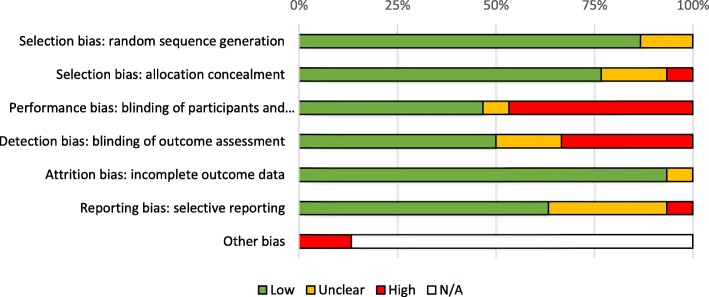
Risk of bias based on authors’ judgement about each risk of bias item expressed as a percentage of all included trials

**Fig. 3:**
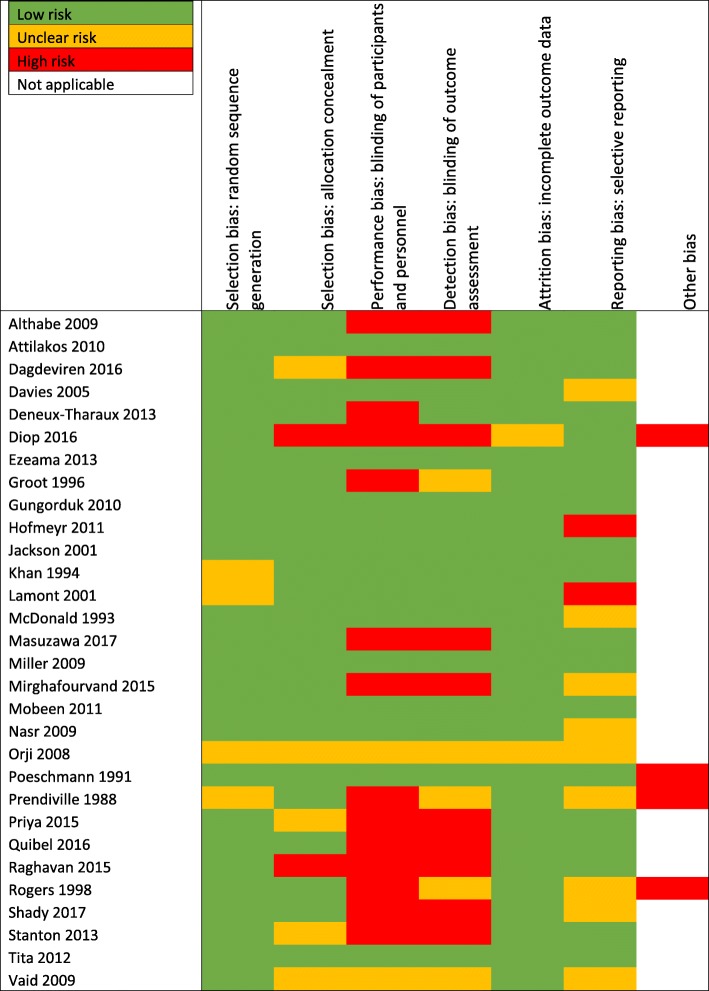
Risk of bias based on authors’ judgement for each included trial

Risk of bias assessment summaries are shown in Figs. 2 and 3. Individual risk of bias assessments can be found in Additional file 1.

The risk of bias was assessed independently by LH and AW for 14 trials [10–23]. Six assessments [24–29] were produced during a review by Gallos et al. [30]. The risk of bias assessments for the Althabe 2009 [31] and Deneux-Tharaux 2013 [32] trials were completed during a review by Hofmeyr, Mshweshwe and Gülmezoglu [33]. Oladapo, Okusanya and Abalos [34] completed a risk of bias assessment for the Dagdeviren 2016 [35] trial. The risk of bias for the Jackson 2001 [9] trial was completed during the review by Soltani, Hutchon and Poulose [36]. Westhoff, Cotter and Tolosa [37] carried out a risk of bias assessment for the Poeschmann 1991 [38] trial. Where appropriate, risk of bias assessments from multiple sources were used to produce a single risk of bias for the current review. Risk of bias assessments for the Stanton 2013 [39] trial were completed by Gallos et al. [30] and Pantja et al. [40]. The Rogers 1998 [41] and Prendiville 1988 [42] risk of bias assessments were completed by Begley et al. [43] and Gallos et al. [30]. The Groot 1996 [44] trial risk of bias was completed by Gallos et al. [30], Liabsuetrakul et al. [45] and Westhoff, Cotter and Tolosa [37]. The Attilakos 2010 [46] risk of bias was assessed by two previous reviews, Gallos et al. [30] and Su, Chong and Samuel [47].

Selection bias was judged to be low risk in 87% of trials for random sequence generation and for 77% of trials for allocation concealment. Four trials were considered to have an unclear risk of selection bias as the methods to describe adequate random sequence generation were not reported or inadequately described [14, 15, 28, 42]. There was an unclear risk for five trials [19, 28, 29, 35, 39] concerning allocation concealment as it was not reported appropriately. The Diop 2016 [11] and Raghavan 2015 [21] trials were cluster randomised trials meaning allocation would have been known in advance so were judged to be high risk for inadequate allocation concealment.

Performance bias was low risk in 47% of trials, unclear risk in 7% and high risk in 47%. Detection bias showed similar results with 50% of trials deemed low risk, 17% unclear and 33% high risk. The large number of trials judged to be unclear or high risk for performance and detection bias were commonly due to difficult blinding participants and personnel to interventions due to the differences in administration [11, 16, 19, 21, 22, 31, 32, 35, 39, 41, 42, 44], unclear reporting of methods [28, 29] and a likelihood that blinding could be broken [18, 20]. The Deneux-Tharaux 2013 [32] and Groot 1996 [44] trials were judged to be high risk of performance bias but low and unclear risk of detection bias, respectively, due to the use of objective measurements for the primary outcome.

Attrition bias was low risk in 93% of trials. The Orji 2008 [28] trial was considered to have an unclear risk of attrition bias as the authors did not report attrition or any incomplete data. The Diop 2016 [12] trial was also considered to have unclear risk as the attrition rates in the misoprostol and oxytocin arms, 27.5 and 22.5% respectively, were high but the authors stated that all women given an intervention were followed up and were included in the analysis. Reporting bias was low risk in 63%, unclear in 30% and high risk in 7%. For eight trials deemed to have unclear risk of reporting bias, the protocol was not published or could not be accessed [10, 18, 25, 27–29, 41, 42]. The Shady 2017 [22] trial was judged to have an unclear risk of reporting bias as the definition of PPH was unclear. The protocol for the Hofmeyr 2011 [13] trial stated the rates of transfusion and haemoglobin < 8 g/dl after 24 h would be reported as secondary outcomes. However, this was not reported in the published trial results and was therefore judged to be high risk of selective reporting. The Lamont 2001 [15] trial did not report all expected outcomes such as additional surgery and transfusion rates so was also considered to be high risk.

Other bias was assessed on an individual basis and included a high risk of bias for early termination of the Poeschmann 1991 [38] and Rogers 1998 [41] trials. The Prendiville 1988 [42] trial was deemed to have a high risk of bias due to a smaller sample size and a protocol change after five months. The data of women included before the protocol change was still included within the final analysis. The Diop 2016 [11] trial changed the huts receiving the misoprostol or oxytocin intervention after initiation of the trial which has been considered to have a high risk of bias.

### Primary outcomes

#### Proportion analysis for minor PPH grades

Overall, there were 28 trials reporting the proportion of women with a minor PPH. **Grade 1** included 11 trials and had a random effects pooled proportion of 0.10 (95% CI = 0.042 to 0.18), with high heterogeneity (*p* < 0.0001; *I*^*2*^ = 99.1%; 95% CI = 98.9 to 99.2%) (Additional file 2: Figure S1) [12, 13, 17–19, 21, 22, 26, 28, 38, 44]. **Grade 2** included four trials and had a random effects pooled proportion of 0.15 (95% CI = 0.067 to 0.26), with high heterogeneity (*p* < 0.0001; *I*^*2*^ of 96.6%; 95% CI = 94.6 to 97.7%) (Additional file 2: Figure S2) [16, 24, 35, 41]. **Grade 3** included two trials and had a random effects pooled proportion of 0.14 (95% CI = 0.095 to 0.18), with medium heterogeneity (*p* = 0.07; *I*^*2*^ = 69.6%) (Additional file 2: Figure S3) [29, 42]. **Grade 4** included 11 trials and had a random effects pooled proportion of 0.10 (95% CI = 0.072 to 0.14), with high heterogeneity (*p* < 0.0001; *I*^*2*^ = 98.1%; 95% CI = 97.8 to 98.4%) (Additional file 2: Figure S4) [9, 10, 14, 15, 20, 23, 25, 27, 31, 32, 39]. There were no trials included in grade 5 for minor PPH.

**Fig. 4:**
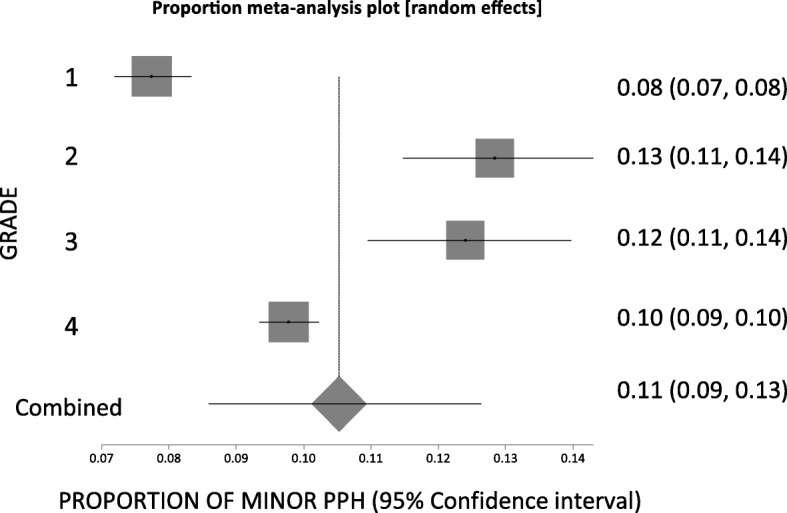
Proportion meta-analysis box plot for overall minor postpartum haemorrhage (PPH) data from graded trials on prophylactic PPH interventions. There is no significant difference in the proportion of PPH across the minor PPH grades (Kruskal-Wallis: T = 0.92, *p* = 0.82). PPH – postpartum haemorrhage

The pooled proportion across all grades for minor PPH was 0.11 (95% CI = 0.086 to 0.13) and there was high heterogeneity across the grades (*p* < 0.0001; *I*^*2*^ = 96.1%; 95% CI = 93.4 to 97.4%) (Fig. 4). Kruskal-Wallis analysis showed no statistically significant difference in minor PPH rates between the different allocated grades (T = 0.92, *p* = 0.82), and no significant difference with an all pairwise comparison of the individual grades.

#### Proportion analysis for major PPH grades

Overall, there were 21 trials reporting results for the proportion of participants with major PPH. **Grade 1** included six trials and had a random effects pooled proportion of 0.033 (95% CI = 0.011 to 0.066), with high heterogeneity across the trials (*p* < 0.0001; *I*^*2*^ = 93.8%; 95% CI = 89.8 to 95.8%) (Additional file 3: Figure S1) [12, 13, 18, 26, 38, 44]. **Grade 2** included three trials and had a random pooled proportion of 0.033 (95% CI 0.012 to 0.063), with high heterogeneity (*p* = 0.004; *I*^*2*^ = 81.9%; 95%CI = 0 to 92.3%) (Additional file 3: Figure S2) [16, 35, 41]. **Grade 3** included one trial with a proportion of 0.020 (95% CI = 0.014 to 0.027) (Additional file 3: Figure S3) [42]. **Grade 4** included 10 trials and had a random effects pooled proportion of 0.035 (95% CI = 0.015 to 0.064), with high heterogeneity (*p* < 0.0001; *I*^*2*^ = 98.5%; 95% CI = 98.3 to 98.8%) (Additional file 3: Figure S4) [9–11, 14, 15, 20, 25, 31, 32, 39]. **Grade 5** included one trial with a proportion of 0.049 (95% CI = 0.030 to 0.073) (Additional file 3: Figure S5) [46].

**Fig. 5:**
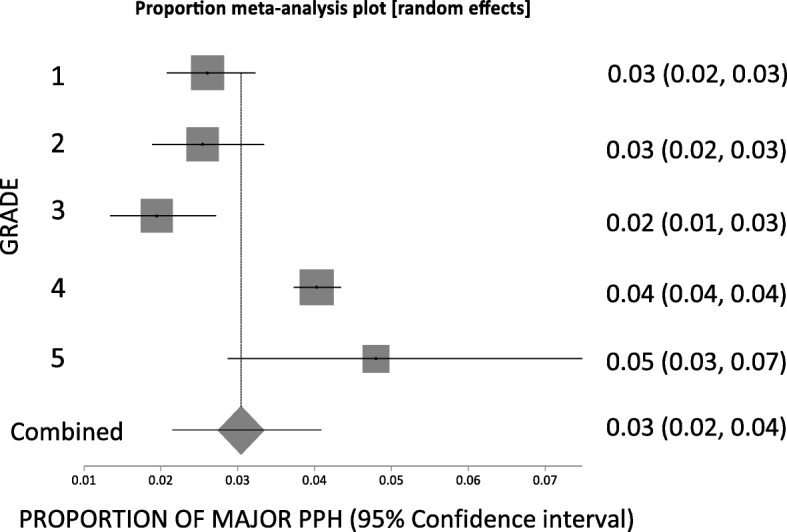
Proportion meta-analysis for overall major postpartum haemorrhage (PPH) data from graded trials on prophylactic PPH interventions. There is no significant difference in the proportion of PPH across the major PPH grades (Kruskal-Wallis: T = 0.91, *p* = 0.92). PPH – postpartum haemorrhage

The pooled proportion across all grades for major PPH was 0.030 (95% CI = 0.022 to 0.041) with high heterogeneity across the grades (p < 0.0001; *I*^*2*^ = 90.8%; 95% CI = 81 to 94.4%) (Fig. 5). Kruskal-Wallis analysis showed that there is no statistically significant difference in major PPH rates between the different grades (T = 0.91, *p* = 0.92), and no significant difference with an all pairwise comparison of the individual grades.

### Secondary outcomes

#### Methods comparison for minor PPH

**Fig. 6:**
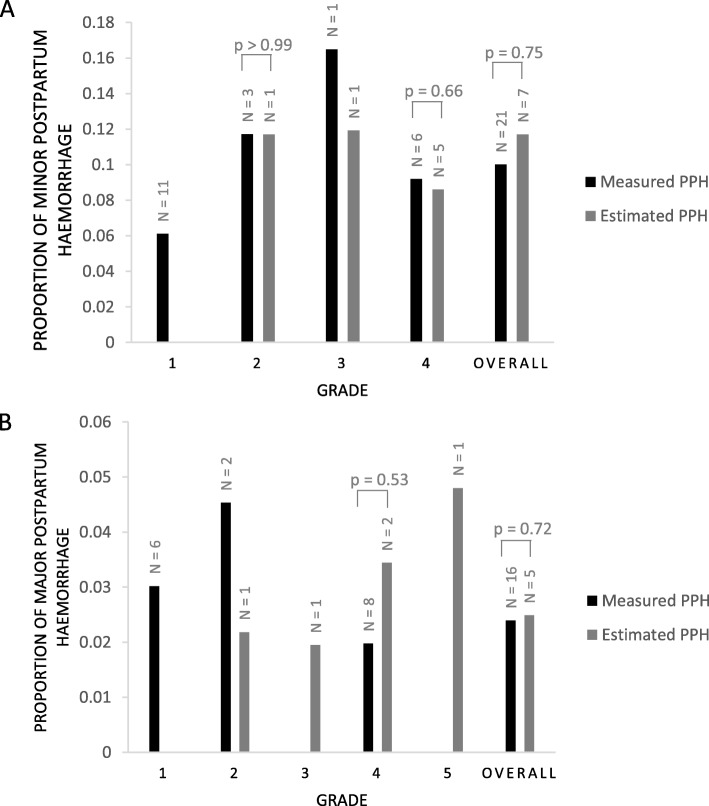
Comparison of methods for determining blood loss in trials for minor and major PPH. **a** Overall, there was no difference in the rates of minor PPH between trials using a measurement of blood loss and those using an estimation (Mann-Whitney U = 67, *p* = 0.75). **b** There was no difference in the rates of major PPH between trials using a measurement of blood loss and those using an estimation (Mann-Whitney U = 35, *p* = 0.72). PPH – postpartum haemorrhage

Seven trials for minor PPH collected blood loss data based on an estimated volume (median rate = 0.12), whereas, 21 trials used a measurement of blood loss (median rate = 0.10) (Fig. 6a). Measurement of blood loss included gravimetric measurement, BRASS-V drape collection, haemoglobin or haematocrit measurement and the traditional measurement of weighing blood-soaked items. Overall, there was no significant difference in the rates of minor PPH between the methods (Mann-Whitney U = 67, p (two-tailed) = 0.75). All trials within **grade 1** used a measurement to determine minor PPH (median rate = 0.061). There was no significant difference in the rates of minor PPH between measured (*n* = 3; median rate = 0.12) or estimated (*n* = 1; median rate = 0.12) methods for **grade 2** trials (Mann-Whitney U = 2, p (two-tailed) > 0.99). **Grade 3** contained two trials, one used a measurement (median rate = 0.17) and one used an estimate of blood loss (median rate = 0.12). There was no significant difference in the rates of minor PPH between measured (*n* = 6, median rate = 0.092) and estimated blood loss (*n* = 5, median rate = 0.086) for **grade 4** trials (Mann-Whitney U = 12, p (two-tailed) = 0.66).

#### Methods comparison for major PPH

For major PPH data, five trials reported data based on estimated blood loss (median rate = 0.024) and 16 trials reported data using a measurement (median rate = 0.025). There was no significant difference between the methods of determining the rate of PPH (Mann-Whitney U = 35, p (two-tailed) = 0.72) (Fig. 6b). All six trials within **grade 1** used a measurement of blood loss (n = 6; median rate = 0.030). Within **grade 2**, two trials used a measurement (median rate = 0.045) and one trial used an estimation (median rate = 0.022). There was one trial in **grade 3** which used an estimate to determine PPH rate (median rate = 0.019). In **grade 4**, there was no significant difference between the measured (*n* = 8; median rate = 0.020) and estimated (*n* = 2; median rate = 0.034) groups for the rate of major PPH (Mann-Whitney U = 5, p (two tailed) = 0.53). **Grade 5** contained one trial which used a method of estimation to determine the rate of PPH (median rate = 0.048).

### Post-hoc analysis

#### Rate of PPH vs. % operative births

**Fig. 7:**
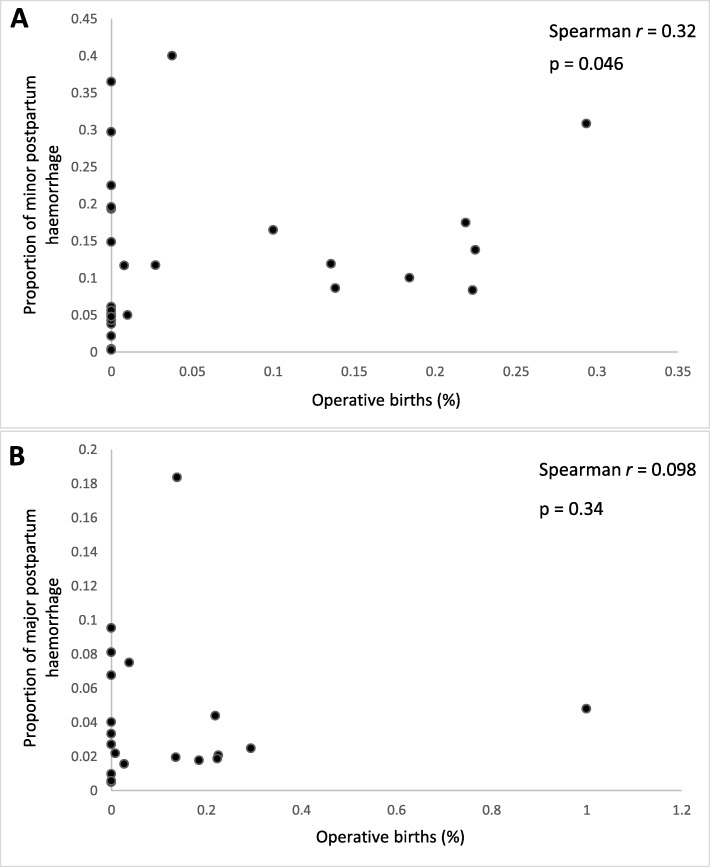
Post hoc analysis of postpartum haemorrhage (PPH) vs. the percentage of operative births (operative vaginal and caesarean section) **a)** The rate of minor PPH significantly correlates with the percentage of operative births (Spearman rho = 0.32, *p* = 0.046). **b** The rate of major PPH did not correlate with the percentage of operative births (Spearman rho = 0.098, *p* = 0.34). PPH – postpartum haemorrhage

Based on the results of post hoc analysis, the percentage of operative births in a study and the rate of minor PPH are significantly correlated (Spearman *r* = 0.32) (Fig. 7a). However, there is no correlation between the percentage of operative births and the rate of major PPH (Spearman *r* = 0.098, p (two-tailed) = 0.34) (Fig. 7b).

## Discussion

This review compares PPH rates in trials of prophylaxis according to the participant inclusion criteria. Best practice recommendations for prophylaxis are based mainly on the evidence from RCTs, which largely only include low risk women who have, by definition, lower rates of haemorrhage. Trials focussing on PPH tend not to include women with more complex births, and therefore, current guidance may not address the target population [4, 48, 49]. Bais et al. suggest that the high PPH rate in their study could be due to their inclusion of high risk women, indicating inclusion criteria impacts reported trial rate [48]. The rates observed in trials of intervention often do not correlate with the WHO estimates of global prevalence. Weeks and Nielson argue that this is the result of widespread inclusion bias towards low risk women in current literature [4].

In this review, there was a disproportionate number of trials which only included low risk women with 39% (11/30) in **grade 1**, the lowest risk category. Despite this, no differences in minor or major PPH rates were identified between grades, suggesting the exclusion of high risk births from trials does not influence the overall trial rate of PPH. The lack of relationship between trial grade and overall rate could be due the effectiveness of interventions in all women, regardless of risk profile. This could imply that interventions have little or no effect on PPH rates in low risk women but result in lower PPH rates for trials including higher risk participants. If this were correct, it would explain the overall similar rates across all grades. However, this is not supported by 70% (21/30) of included trials reporting non-significant results for their intervention.

Commonly used uterotonics are based on evidence from low risk births which is then generalised to all women [4]. There is little evidence to describe their relationship with bleeding in complex births. Post-hoc analysis on the relationship of PPH with operative births (operative vaginal and caesarean section) identified significant correlation with minor, but nor major, PPH (Spearman *r* = 0.32, *p* = 0.046 v. Spearman *r* = 0.098, *p* = 0.34). Method of birth could have more influence on PPH rate than antenatal risk factors. In this review, a significant correlation was only found with minor PPH. However, this observed correlation is consistent with data from the Liverpool Women’s Hospital, which shows that operative births disproportionately contribute to the incidence of haemorrhages > 2000 ml [4]. In 2009–2013, operative births comprised 39.1% of births but 71.6% of PPH cases. The impact of operative births on PPH rates was also observed in a Scottish study where emergency C sections accounted for 41% of haemorrhages ≥2500 ml but only 15% of total births in Scotland [50]. Despite evidence that women undergoing operative births are at higher risk of haemorrhage, they are often excluded from trial participation. In this review, over half (56.7%) of trials excluded all operative births entirely. There needs to be more weight given to trials which include women with complex births as they are most in need of effective interventions [4].

The impact of visual estimation over objective measurement has been widely described [6, 51–53]. This review did not identify a difference in PPH rates between studies that used estimation and measurement, contrary to many reports of inaccuracies with visual estimation in another review [6]. However, this current review defined measurement as any attempt at objectifying blood loss. This is not concordant with other trials and reviews which consider the traditional method as an estimation, where the loss is calculated from visual estimation and measurement of blood in drainage bottles and gauze [51]. This difference in definition could also explain the unexpectedly low number of trials using estimation (25% of minor and 24% of major PPH).

### Limitations

There was considerable heterogeneity across trials in all grades, for both minor and major PPH, with *I*^*2*^ ranging from 69.6 to 99.1%. This suggests that there may be other factors other than antenatal risk factors and method of birth which would explain the differences in PPH rates in trials of prophylaxis. It would have been useful to identify predictors of rate through multiple regression; however, this review was limited by the inadequately sized data set. The small data set also prevented analysis of both minor and major PPH across all five grades. There was only one included trial in **grade 5**, which reported data for PPH > 1000 ml only. Pooling of data from both arms of the trials is justified as 70% of trials showed no difference in rates. However, an alternative that could be explored is the use of data from the control arm only. As discussed, another limitation of this review was the definition of estimated and measured blood loss, which is inconsistent with other literature, resulting in only a small number of trials being identified as using estimation.

### What is already known on this topic

Postpartum haemorrhage remains a leading cause of maternal death.

There is widespread inclusion bias towards low risk women in trials of intervention.

Operative births contribute to a large number of PPH incidents.

### What this study adds

There is a disproportionate number of studies focussing only on low risk women and births.

Grading trials using antenatal risk factors and method of birth does not explain the variance in PPH rates.

Method of birth could be a more important predictor of PPH rate and hence, more weight should be given to studies focussing on complex births.

## Conclusion

This review showed that antenatal risk factors may not be a significant contributor to the variance in trial rates between studies, but the method of birth could have more impact. Operative vaginal may be an important contributor to PPH but women who give birth this way are often excluded from participating in trials and data supporting interventions in this population is therefore lacking. More focus on these women when conducting trials of intervention would be more appropriate to ensure that current recommendations for complex births are formed using a relevant evidence base.

## Supplementary information

**Additional file 1.** Risk of bias assessment for each of the included trials in detail with author judgment and support for this judgement.

**Additional file 2.** Proportion meta-analysis box plots for the individual grades for minor postpartum haemorrhage.

**Additional file 3.** Proportion meta-analysis box plots for the individual grades for major postpartum haemorrhage.

**Additional file 4.** Table of trials included in “A systematic review of postpartum haemorrhage (PPH) rates in randomized trials of PPH prophylactic interventions to examine the effect of underlying participant PPH risk”.

## Data Availability

All relevant data are given within the manuscript and the supplementary files (Additional file 4 table of trials included in this review).

## Abbreviations

CENTRAL: Cochrane Central Register of Controlled Trials
CI: Confidence interval
NICE: National Institute of Clinical Excellence
PPH: Postpartum haemorrhage
PRISMA: Preferred Reporting Items for Systematic Reviews and Meta-Analysis
RCOG: Royal College of Obstetricians and Gynaecologists
RCT: Randomised controlled trial
WHO: World Health Organisation
