# Supplementary material for: Postpartum haemorrhage (PPH) rates in randomized trials of PPH prophylactic interventions and the effect of underlying participant PPH risk: a meta-analysis
Source: BMC Pregnancy Childbirth. 2020 Feb 13;20:107. doi: 10.1186/s12884-020-2719-3 (PMC7020586; doi:10.1186/s12884-020-2719-3)
Supplement: Supplementary file 1 — Additional file 1. Risk of bias assessment for each of the included trials in detail with author judgment and support for this judgement. [file 12884_2020_2719_MOESM1_ESM.pdf]

Additional File 1 - Individual risk of bias assessments

The following assessments have been conducted with the use of previous Cochrane review assessments;

Althabe 2009

Domain	Review authors judgement	Support for judgement
Random sequence generation (selection bias)	Low risk	Sequence generated at the co-ordinating centre using computer-generated list of numbers with randomly permuted blocks of 4-6 in a 1:1 ratio
Allocation concealment (selection bias)	Low risk	Use of sequentially numbered opaque sealed envelopes. When a woman is about to deliver, next numbered envelope was opened
Blinding of participants and personnel (performance bias)<br>All outcomes	High risk	**Participant**: not blinded<br>**Clinician**: not blinded
Blinding of outcome assessment (detection bias)<br>All outcomes	High risk	**Outcome assessor**: not blinded
Incomplete outcome data (attrition bias)<br>All outcomes	Low risk	Only 5 women not included in analysis
Selective reporting (reporting bias)	Low risk	No indication of selective reporting

Hofmeyr GJ, Mshweshwe NT, Gülmezoglu AM. Controlled cord traction for the third stage of labour. Cochrane Database of Systematic Reviews 2015(1): Art. No.: CD008020. DOI: 10.1002/14651858.CD008020.pub.

Attilakos 2010

Domain	Review authors judgement	Support for judgement
Random sequence generation (selection bias)	Low risk	The randomisation sequence (1:1 ratio—blocks of 10, no stratification) was generated by computer.
Allocation concealment (selection bias)	Low risk	The preparation of the ampoules was undertaken by DHP Ltd. (Powys, UK). All boxes and ampoules were identically labelled. The random allocation sequence was not known to the investigators until the study had finished and the analysis was started.
Blinding of participants and personnel (performance bias) All outcomes	Low risk	Study participants and caregivers were blinded to treatment allocations.
Blinding of outcome assessment (detection bias) All outcomes	Low risk	Assessors were blinded to treatment allocations.
Incomplete outcome data (attrition bias) All outcomes	Low risk	Data were collected completely from all randomised study participants.
Selective reporting (reporting bias)	Low risk	The study report matches the study protocol that was registered prospectively (EudraCT 2005-002812-94).

Gallos ID, Papadopoulou A, Man R, Athanasopoulos N, Tobias A, Price MJ, et al. Uterotonic agents for preventing postpartum haemorrhage: a network meta-analysis. Cochrane Database of Systematic Reviews 2018(12): Art No.: CD011689. DOI: 10.1002/14651858.CD011689.pub3.

Su LL, Chong YS, Samuel M. Carbetocin for preventing postpartum haemorrhage. Cochrane Database of Systematic Reviews 2012(4): Art. No.: CD005457. DOI: 10.1002/14651858.CD005457.pub4.

Dagdeviren 2016

Domain	Review authors judgement	Support for judgement
Random sequence generation (selection bias)	Low risk	Reported that a random number table was used to determine the sequence for randomisation
Allocation concealment (selection bias)	Unclear risk	The way women were allocated to groups at the point of randomisation was not clear. It was stated that women were divided into 2 “equal” groups and randomisation was at the point when delivery was imminent.
Blinding of participants and personnel (performance bias) All outcomes	High risk	Staff providing care and making clinical decisions about interventions would be aware of which intervention women received. The study protocol stated that there was no attempt to mask treatment from women and personnel.
Blinding of outcome assessment (detection bias) All outcomes	High risk	Outcomes would be recorded by staff aware of allocation, however, objective outcomes such as haemoglobin would have a lower risk of bias.
Incomplete outcome data (attrition bias) All outcomes	Low risk	No loss to follow up
Selective reporting (reporting bias)	Low risk	This was a registered study and expected outcomes were reported. Incidence of prolonged 3rd stage not reported but duration was reported as a mean.

Oladapo OT, Okusanya BO, Abalos E. Intramuscular versus intravenous prophylactic oxytocin for the third stage of labour. Cochrane Database of Systematic Reviews 2018(9): Art. No.: CD009332. DOI: 10.1002/14651858.CD009332.pub3

Deneux-Tharaux 2013

Domain	Review authors judgement	Support for judgement
Random sequence generation (selection bias)	Low risk	Randomisation was done through an automated web-based system, stratified by centre and balanced in blocks of four
Allocation concealment (selection bias)	Low risk	Centrally through an automated web-based system, which ensured allocation concealment
Blinding of participants and personnel (performance bias) All outcomes	High risk	Not possible to blind due to interventions
Blinding of outcome assessment (detection bias) All outcomes	Low risk	Blinding not possible, but primary outcome objective measurement of blood loss
Incomplete outcome data (attrition bias) All outcomes	Low risk	After randomisation and before delivery, 294 (6.8%) women became ineligible because an intrapartum caesarean was performed, and three others declined to participate. Women who underwent caesarean section were included in the analysis for outcomes where this was possible
Selective reporting (reporting bias)	Low risk	No indication of selective reporting

Hofmeyr GJ, Mshweshwe NT, Gülmezoglu AM. Controlled cord traction for the third stage of labour. Cochrane Database of Systematic Reviews 2015(1): Art. No.: CD008020. DOI: 10.1002/14651858.CD008020.pub.

Ezeama 2013

Domain	Review authors judgement	Support for judgement
Random sequence generation (selection bias)	Low risk	"Computer-generated randomization" was used
Allocation concealment (selection bias)	Low risk	A person uninvolved with the study prepared the study drugs. The labels on the ampoules (which were similar in size and colour) were removed and the ampoules were placed in opaque sealed envelopes.
Blinding of participants and personnel (performance bias)<br>All outcomes	Low risk	"A person uninvolved with the study prepared the study drugs… The labels on the ampoules (which were similar in size and colour) were removed and the ampoules were placed in opaque sealed envelopes, such that only the computer-generated randomisation numbers on the envelopes were available to identify the study drug during unblinding."
Blinding of outcome assessment (detection bias)<br>All outcomes	Low risk	Assessors were blinded to treatment allocations.
Incomplete outcome data (attrition bias)<br>All outcomes	Low risk	Data were collected completely from all randomised study participants.
Selective reporting (reporting bias)	Low risk	The study protocol was registered (PACTR 201105000292708).

Gallos ID, Papadopoulou A, Man R, Athanasopoulos N, Tobias A, Price MJ, et al. Uterotonic agents for preventing postpartum haemorrhage: a network meta-analysis. Cochrane Database of Systematic Reviews 2018(12): Art No.: CD011689. DOI: 10.1002/14651858.CD011689.pub3.

Groot 1996

Domain	Review authors judgement	Support for judgement
Random sequence generation (selection bias)	Low risk	Randomisation was achieved using a computer-generated random list.
Allocation concealment (selection bias)	Low risk	Investigators used identical study boxes. Care was taken that no difference could be seen or heard between the packages of the ergometrine/placebo tablets and the oxytocin ampoules.
Blinding of participants and personnel (performance bias) All outcomes	High risk	The study was "double-blind" with placebo to match ergometrine treatment, but "to allow comparison with a standard prophylactic regimen a third group receiving the standard intramuscular oxytocin was added, but for obvious reasons this could not be conducted in a blind manner."
Blinding of outcome assessment (detection bias) All outcomes	Unclear risk	Assessor blinding was not reported, however, objective blood loss measurements were used.
Incomplete outcome data (attrition bias) All outcomes	Low risk	"4 women with exclusion criteria were entered erroneously. They are considered as non-participants."
Selective reporting (reporting bias)	Low risk	It is clear that the published reports include all expected outcomes specified.

Gallos ID, Papadopoulou A, Man R, Athanasopoulos N, Tobias A, Price MJ, et al. Uterotonic agents for preventing postpartum haemorrhage: a network meta-analysis. Cochrane Database of Systematic Reviews 2018(12): Art No.: CD011689. DOI: 10.1002/14651858.CD011689.pub3.

Liabsuetrakul T, Choobun T, Peeyananjarassri K, Islam QM. Prophylactic use of ergot alkaloids in the third stage of labour. Cochrane Database of Systematic Reviews 2018(6): Art. No.: CD005456. DOI: 10.1002/14651858.CD005456.pub3.

Westhoff G, Cotter AM, Tolosa JE. Prophylactic oxytocin for the third stage of labour to prevent postpartum haemorrhage. Cochrane Database of Systematic Reviews 2013(10): Art. No.: CD001808. DOI: 10.1002/14651858.CD001808.pub2.

Jackson 2001

Domain	Review authors judgement	Support for judgement
Random sequence generation (selection bias)	Low risk	"A computer-generated randomization was used to dictate which labeled vial in each pair would contain oxytocin and which would contain only normal saline.
Allocation concealment (selection bias)	Low risk	Allocation was done sequentially, "each participant was assigned… on the basis of the order in which she was enrolled"
Blinding of participants and personnel (performance bias) All outcomes	Low risk	"Neither patients nor clinicians knew which infusion contained oxytocin"
Blinding of outcome assessment (detection bias) All outcomes	Low risk	As above
Incomplete outcome data (attrition bias) All outcomes	Low risk	Data was available for all participants, for all outcomes
Selective reporting (reporting bias)	Low risk	No specific reporting bias was identified.

Soltani H, Hutchon DR, Poulose TA. Timing of prophylactic uterotonics for the third stage of labour after vaginal birth. Cochrane Database of Systematic Reviews 2010(8): Art. No.: CD006173. DOI: 10.1002/14651858.CD006173.pub2.

McDonald 1993

Domain	Review authors judgement	Support for judgement
Random sequence generation (selection bias)	Low risk	The ampoules were numbered by Sandoz using simple randomisation. There was no blocking or prognostic stratification.
Allocation concealment (selection bias)	Low risk	The ampoules were numbered by third party (Sandoz).
Blinding of participants and personnel (performance bias) All outcomes	Low risk	Delivery attendants were blinded to treatment allocations.
Blinding of outcome assessment (detection bias) All outcomes	Low risk	Assessors were blinded to treatment allocations.
Incomplete outcome data (attrition bias) All outcomes	Low risk	"All women allocated to receive a drug were included in that group, excluding only the 14 women for whom drug allocation was not recorded."
Selective reporting (reporting bias)	Unclear risk	The protocol of the study was unavailable for verification.

Gallos ID, Papadopoulou A, Man R, Athanasopoulos N, Tobias A, Price MJ, et al. Uterotonic agents for preventing postpartum haemorrhage: a network meta-analysis. Cochrane Database of Systematic Reviews 2018(12): Art No.: CD011689. DOI: 10.1002/14651858.CD011689.pub3.

.

Mobeen 2011

Domain	Review authors judgement	Support for judgement
Random sequence generation (selection bias)	Low risk	A computer-generated random code in blocks of 6 was maintained by Gynuity Health Projects in New York and not revealed until data collection and cleaning were completed.
Allocation concealment (selection bias)	Low risk	Study medication was packed in numbered colour-coded boxes by Gynuity Health Projects in New York.
Blinding of participants and personnel (performance bias) All outcomes	Low risk	"Both women and TBAs were blinded to study assignment."
Blinding of outcome assessment (detection bias) All outcomes	Low risk	Assessors were blinded to treatment allocations.
Incomplete outcome data (attrition bias) All outcomes	Low risk	"Invalid blood loss measures, which mainly occurred when monitoring visits were not possible because of poor weather conditions, were excluded from our analysis."
Selective reporting (reporting bias)	Low risk	The study report matches the study protocol that was registered (ClinicalTrials.gov NCT00120237).

Gallos ID, Papadopoulou A, Man R, Athanasopoulos N, Tobias A, Price MJ, et al. Uterotonic agents for preventing postpartum haemorrhage: a network meta-analysis. Cochrane Database of Systematic Reviews 2018(12): Art No.: CD011689. DOI: 10.1002/14651858.CD011689.pub3.

Nasr 2009

Domain	Review authors judgement	Support for judgement
Random sequence generation (selection bias)	Low risk	Treatment was allocated by a computer-generated random allocation system created at the Statistics Unit of Assiut University Hospital.
Allocation concealment (selection bias)	Low risk	Allocation codes were placed in sealed, opaque, consecutively-numbered envelopes.
Blinding of participants and personnel (performance bias) All outcomes	Low risk	The study was "double-blind": active treatments and placebo treatments were "identical-looking."
Blinding of outcome assessment (detection bias) All outcomes	Low risk	Assessors were blinded to treatment allocations.
Incomplete outcome data (attrition bias) All outcomes	Low risk	Data were collected completely from all randomised study participants.
Selective reporting (reporting bias)	Unclear risk	The protocol of the study was unavailable for verification.

Gallos ID, Papadopoulou A, Man R, Athanasopoulos N, Tobias A, Price MJ, et al. Uterotonic agents for preventing postpartum haemorrhage: a network meta-analysis. Cochrane Database of Systematic Reviews 2018(12): Art No.: CD011689. DOI: 10.1002/14651858.CD011689.pub3.

Orji 2008

Domain	Review authors judgement	Support for judgement
Random sequence generation (selection bias)	Unclear risk	Randomisation sequence generation was not reported.
Allocation concealment (selection bias)	Unclear risk	Allocation was done by sealed sequentially-numbered envelopes.
Blinding of participants and personnel (performance bias) All outcomes	Unclear risk	Blinding (of study participants and caregivers) was not reported.
Blinding of outcome assessment (detection bias) All outcomes	Unclear risk	Assessor blinding was not reported.
Incomplete outcome data (attrition bias) All outcomes	Unclear risk	The study authors did not mention any incomplete outcome data.
Selective reporting (reporting bias)	Unclear risk	The protocol of the study was unavailable for verification, but not all of the outcomes projected by methodological descriptions were reported as results in the study report (cases of transfusion and PPH at least 1000 mL were omitted).

Gallos ID, Papadopoulou A, Man R, Athanasopoulos N, Tobias A, Price MJ, et al. Uterotonic agents for preventing postpartum haemorrhage: a network meta-analysis. Cochrane Database of Systematic Reviews 2018(12): Art No.: CD011689. DOI: 10.1002/14651858.CD011689.pub3.

Poeschmann 1991

Domain	Review authors judgement	Support for judgement
Random sequence generation (selection bias)	Low risk	A random allocation list was set prior to the trial
Allocation concealment (selection bias)	Low risk	"The hospital pharmacy supplied numbered boxes… allocation of the boxes was by order of entry" demonstrates adequate concealment
Blinding of participants and personnel (performance bias)	Low risk	Blinding was achieved by using "a nurse not working in the labour room" preparing the injection
Blinding of outcome assessment (detection bias)	Low risk	As above
Incomplete outcome data (attrition bias)	Low risk	All outcomes are reported
Selective reporting (reporting bias)	Low risk	All expected outcomes are reported
Other bias	High risk	Trial was stopped early due to organisational issues

Westhoff G, Cotter AM, Tolosa JE. Prophylactic oxytocin for the third stage of labour to prevent postpartum haemorrhage. Cochrane Database of Systematic Reviews 2013(10): Art. No.: CD001808. DOI: 10.1002/14651858.CD001808.pub2.

Prendiville 1988

Domain	Review authors judgement	Support for judgement
Random sequence generation (selection bias)	Unclear risk	Randomisation sequence generation was not reported.
Allocation concealment (selection bias)	Low risk	Investigators used sequentially-numbered, opaque, sealed envelopes.
Blinding of participants and personnel (performance bias) All outcomes	High risk	Study participants and caregivers were not blinded to treatment allocations.
Blinding of outcome assessment (detection bias) All outcomes	Unclear risk	Assessor blinding was not reported.
Incomplete outcome data (attrition bias) All outcomes	Low risk	Data were collected completely from all randomised study participants.
Selective reporting (reporting bias)	Unclear risk	The protocol of the study was unavailable for verification.
Other bias	High risk	The protocol was modified after 5 months due to high blood loss in the expectant group but data from the initial 5 months were included in the analysis. Sample size was supposed to be 3900 but stopped at 1695. 30 women in the control group gave a late maternal refusal, whereas only 1 in the experimental group did so. The outcomes of these women are included in analysis.

Begley CM, Gyte GML, Devane D, McGuire W, Weeks A. Active versus expectant management for women in the third stage of labour. Cochrane Database of Systematic Reviews 2015(3): Art No.: CD007412. DOI: 10.1002/14651858.CD007412.pub4.

Gallos ID, Papadopoulou A, Man R, Athanasopoulos N, Tobias A, Price MJ, et al. Uterotonic agents for preventing postpartum haemorrhage: a network meta-analysis. Cochrane Database of Systematic Reviews 2018(12): Art No.: CD011689. DOI: 10.1002/14651858.CD011689.pub3.

Rogers 1998

Domain	Review authors judgement	Support for judgement
Random sequence generation (selection bias)	Low risk	Variable sized balanced blocks "...randomisation envelopes were prepared in advance..." in an external academic unit - National Perinatal Epidemiology Unit, Oxford.
Allocation concealment (selection bias)	Low risk	Sequentially numbered, opaque, sealed envelopes stored on the ward. Entry to the trial occurred when an envelope was opened.
Blinding of participants and personnel (performance bias) All outcomes	High risk	Not possible to blind participants and personnel.
Blinding of outcome assessment (detection bias) All outcomes	Unclear risk	Partly blinded. The technicians who did antenatal and postnatal blood tests were unaware of allocation.
Incomplete outcome data (attrition bias) All outcomes	Low risk	Data available on 1507 out of 1512 at discharge (less than 0.5% attrition, approximately equal losses in both groups). At 6 weeks follow-up less than 5% attrition.
Selective reporting (reporting bias)	Unclear risk	Both significant and non-significant results presented but we have not been able to check the trial protocol. Most as above reported plus others. Only a few neonatal outcomes reported.
Other bias	High risk	Sample size was supposed to be 2000 but interim analysis showed a higher postpartum haemorrhage rate than expected so was stopped early at 1500.

Begley CM, Gyte GML, Devane D, McGuire W, Weeks A. Active versus expectant management for women in the third stage of labour. *Cochrane Database of Systematic Reviews* 2015(3): Art No.: CD007412. DOI: 10.1002/14651858.CD007412.pub4.

Gallos ID, Papadopoulou A, Man R, Athanasopoulos N, Tobias A, Price MJ, et al. Uterotonic agents for preventing postpartum haemorrhage: a network meta-analysis. Cochrane Database of Systematic Reviews 2018(12): Art No.: CD011689. DOI: 10.1002/14651858.CD011689.pub3.

Stanton 2013

Domain	Review authors judgement	Support for judgement
Random sequence generation (selection bias)	Low risk	The 52 CHOs were randomly allocated equally to either the intervention or the control group; this allocation was stratified by both district and distance (at least 10 km, or less than 10 km) to emergency obstetric care. The randomisation sequence was determined using Stata (version 12).
Allocation concealment (selection bias)	Unclear risk	Allocation concealment was not reported.
Blinding of participants and personnel (performance bias) All outcomes	High risk	"The random allocation was not masked."
Blinding of outcome assessment (detection bias) All outcomes	High risk	Assessors were not blinded to treatment allocations.
Incomplete outcome data (attrition bias) All outcomes	Low risk	"7 and 9 enrolled women in the oxytocin and control arms, respectively, lacked a blood-loss measure."
Selective reporting (reporting bias)	Low risk	The study report matches the study protocol that was registered (ClinicalTrials.gov NCT01108289).

Gallos ID, Papadopoulou A, Man R, Athanasopoulos N, Tobias A, Price MJ, et al. Uterotonic agents for preventing postpartum haemorrhage: a network meta-analysis. Cochrane Database of Systematic Reviews 2018(12): Art No.: CD011689. DOI: 10.1002/14651858.CD011689.pub3.

Pantoja T, Abalos E, Chapman E, Vera C, Serrano VP. Oxytocin for preventing postpartum haemorrhage (PPH) in non-facility birth settings. Cochrane Database of Systematic Reviews 2016(4): Art. No.: CD011491. DOI: 10.1002/14651858.CD011491.pub2.

Vaid 2009

Domain	Review authors judgement	Support for judgement
Random sequence generation (selection bias)	Low risk	Treatment was allocated by a computer-generated random number.
Allocation concealment (selection bias)	Unclear risk	Allocation concealment was not reported.
Blinding of participants and personnel (performance bias)<br>All outcomes	Unclear risk	Blinding (of study participants and caregivers) was unclear.
Blinding of outcome assessment (detection bias)<br>All outcomes	Unclear risk	Assessor blinding was not reported.
Incomplete outcome data (attrition bias)<br>All outcomes	Low risk	Data were collected completely from all randomised study participants.
Selective reporting (reporting bias)	Unclear risk	The protocol of the study was unavailable for verification.

Gallos ID, Papadopoulou A, Man R, Athanasopoulos N, Tobias A, Price MJ, et al. Uterotonic agents for preventing postpartum haemorrhage: a network meta-analysis. Cochrane Database of Systematic Reviews 2018(12): Art No.: CD011689. DOI: 10.1002/14651858.CD011689.pub3.

The following assessments have been conducted by LH, and have been checked by AW;

Davies 2005

Domain	Review authors judgement	Support for judgement
Random sequence generation (selection bias)	Low risk	Randomisation was carried out using a random numbers table
Allocation concealment (selection bias)	Low risk	"sealed, numbered packages"
Blinding of participants and personnel (performance bias)	Low risk	A placebo was used which looked the same as the intervention used in the opposite arm, so both personnel and participants were blinded
Blinding of outcome assessment (detection bias)	Low risk	As above
Incomplete outcome data (attrition bias)	Low risk	No attrition
Selective reporting (reporting bias)	Unclear risk	Protocol not seen

Diop 2016

Domain	Review authors judgement	Support for judgement
Random sequence generation (selection bias)	Low risk	"Maternity huts with auxiliary midwives located 3–21 km from the closest referral centre were randomly assigned (1:1) by staff at Gynuity Health Projects"
Allocation concealment (selection bias)	High risk	As a cluster randomised trial, allocation would be known in advance.
Blinding of participants and personnel (performance bias)	High risk	Blinding was not possible due to differences in administration
Blinding of outcome assessment (detection bias)	High risk	As above. Assessors would not have been blinded
Incomplete outcome data (attrition bias)	Unclear risk	After randomisation, there was an attrition rate of 27.5% in the misoprostol group and 22.5% in the oxytocin group. However, all women given the interventions were followed up and data was analysed
Selective reporting (reporting bias)	Low risk	All outcomes were previously discussed and are the same as the published registration (NCT01713153)
Other bias	High risk	Selected huts in the oxytocin and misoprostol groups were changed after initiation of the trial

Gungorduk 2010

Domain	Review authors judgement	Support for judgement
Random sequence generation (selection bias)	Low risk	Random number table was used
Allocation concealment (selection bias)	Low risk	"Infusion bags were prepared in accordance with randomization and labeled as bag A (oxytocin group), which contained 20 international units oxytocin diluted with 26 mL of saline, and bag B (placebo group)"
Blinding of participants and personnel (performance bias)	Low risk	"Providers and patients were blinded to the contents of the bags" as the bags were labelled generically by pharmacists not involved further in the trial. However, labelling A and B would have indicated different interventions.
Blinding of outcome assessment (detection bias)	Low risk	As above
Incomplete outcome data (attrition bias)	Low risk	Loss to follow up equated to less than 10% on those allocated to an intervention
Selective reporting (reporting bias)	Low risk	Outcomes reported as per protocol

Hofmeyr 2011

Domain	Review authors judgement	Support for judgement
Random sequence generation (selection bias)	Low risk	"The random allocation sequence was generated by using computer-generated random numbers and was stratified by country in blocks of 6–8"
Allocation concealment (selection bias)	Low risk	Study packs were selected sequentially as each woman was enrolled and "the study drug packs were prepared, by Gynuity Health Projects. The packs were identical in shape, color, weight, and feel"
Blinding of participants and personnel (performance bias)	Low risk	Packs containing tablets identical in appearance were prepared at a different site, maintaining blinding of personnel and participants
Blinding of outcome assessment (detection bias)	Low risk	As above
Incomplete outcome data (attrition bias)	Low risk	Less than 0.5% loss to follow up. Primary outcome data relevant to this review was available for all participants followed up.
Selective reporting (reporting bias)	High risk	Secondary outcome data for blood transfusion and haemoglobin level <8 g/dL 24 hours after delivery was not reported as described in the published registration (NCT00124540)

Khan 1995

Domain	Review authors judgement	Support for judgement
Random sequence generation (selection bias)	Unclear risk	There is no specific description of the randomisation methods used.
Allocation concealment (selection bias)	Low risk	"opaque sealed envelope"
Blinding of participants and personnel (performance bias)	Low risk	"by the hospital pharmacist who alone was aware of the content of the ampoules"
Blinding of outcome assessment (detection bias)	Low risk	As above
Incomplete outcome data (attrition bias)	Low risk	No attrition after randomisation
Selective reporting (reporting bias)	Low risk	Although no protocol was available, all the expected outcomes have been reported.

Lamont 2001

Domain	Review authors judgement	Support for judgement
Random sequence generation (selection bias)	Unclear risk	The authors state that participants were randomly allocated to an arm but do not explain the randomisation process in full, however, we believe that randomisation was likely adequate
Allocation concealment (selection bias)	Low risk	"randomisation slips were contained in enveloped opened by a person not involved in the trial… who resealed the envelope"
Blinding of participants and personnel (performance bias)	Low risk	Blinding was achieved through preparation of the drug by an independent person not involved in further care of participants or assessments
Blinding of outcome assessment (detection bias)	Low risk	As above.
Incomplete outcome data (attrition bias)	Low risk	No attrition
Selective reporting (reporting bias)	High risk	Not all expected outcomes for this trial have been reported – such as additional surgery and transfusion rates

Masuzawa 2017

Domain	Review authors judgement	Support for judgement
Random sequence generation (selection bias)	Low risk	"Participants were randomly assigned… by use of a web-based randomisation system, with permuted blocks of six and stratified by parity"
Allocation concealment (selection bias)	Low risk	The investigator did not know allocation until the time of placental delivery thus had no prior knowledge
Blinding of participants and personnel (performance bias)	High risk	Blinding not possible due to nature of the intervention
Blinding of outcome assessment (detection bias)	High risk	As above
Incomplete outcome data (attrition bias)	Low risk	Although some women did not receive or discontinued the intervention, all data from randomised participants was analysed
Selective reporting (reporting bias)	Low risk	Outcomes reported as per protocol (UMIN000019834)

Miller 2009

Domain	Review authors judgement	Support for judgement
Random sequence generation (selection bias)	Low risk	"To ensure balanced randomization and to conceal treatment assignment, a computer-generated randomization list with a random block size for each hospital was used. The data coordinating center generated a randomization list stratified by hospital"
Allocation concealment (selection bias)	Low risk	"sequentially numbered sealed opaque envelope out of a locked cabinet in the delivery room"
Blinding of participants and personnel (performance bias)	Low risk	Envelopes were prepared by a member of the research team not involved in patient care. As misoprostol and ZB11 are administered at different times, a placebo was used alongside the allocated intervention to avoid knowledge of the intervention by the clinician or participant
Blinding of outcome assessment (detection bias)	Low risk	Blinding of assessor as above, with objective measurement of the primary outcome (postpartum haemorrhage) used
Incomplete outcome data (attrition bias)	Low risk	Attrition was low as data was available for 960/967 women who had been randomised to the trial (less than 1% attrition)
Selective reporting (reporting bias)	Low risk	Outcomes reported as per protocol (NCT00147420)

Mirghafourvand 2015

Domain	Review authors judgement	Support for judgement
Random sequence generation (selection bias)	Low risk	"...block randomisation, stratified according to number of births (first and second and more childbirths) with block sizes of 4 and 6 and allocation ratio of 1:1."
Allocation concealment (selection bias)	Low risk	"...opaque sequentially numbered sealed packages"
Blinding of participants and personnel (performance bias)	High risk	A nurse not involved directly with participant prepared the drugs so "the data assessor and participants had no knowledge of the study medication." However, it is likely that blinding could have been broken.
Blinding of outcome assessment (detection bias)	High risk	As above
Incomplete outcome data (attrition bias)	Low risk	No attrition
Selective reporting (reporting bias)	Unclear risk	Protocol not seen.

Priya 2015

Domain	Review authors judgement	Support for judgement
Random sequence generation (selection bias)	Low risk	"They were randomized using computer-generated numbers"
Allocation concealment (selection bias)	Unclear risk	There is no specific description of allocation concealment methods
Blinding of participants and personnel (performance bias)	High risk	Blinding not possible due to differences in administration
Blinding of outcome assessment (detection bias)	High risk	As above
Incomplete outcome data (attrition bias)	Low risk	No attrition
Selective reporting (reporting bias)	Low risk	All expected outcomes for this trial type were reported

Quibel 2016

Domain	Review authors judgement	Support for judgement
Random sequence generation (selection bias)	Low risk	"An independent, centralized computer-generated randomization sequence… based on a randomization list established by an independent statitician"
Allocation concealment (selection bias)	Low risk	"To conceal allocation, treatment boxes were sealed and numbered sequentially"
Blinding of participants and personnel (performance bias)	High risk	"The treatment was administered by a research midwife who did not otherwise participate in this trial". However, it is likely that blinding could have been broken.
Blinding of outcome assessment (detection bias)	High risk	As above
Incomplete outcome data (attrition bias)	Low risk	There was no loss to follow up
Selective reporting (reporting bias)	Low risk	As per protocol with an additional post hoc analysis (NCT01113229)

Raghavan 2015

Domain	Review authors judgement	Support for judgement
Random sequence generation (selection bias)	Low risk	"Randomisation was performed by Gynuity Health Projects using a computer-generated random sequence within each stratum.
Allocation concealment (selection bias)	High risk	This was a cluster randomised trial, so allocation will have been known in advance.
Blinding of participants and personnel (performance bias)	High risk	Blinding was not possible
Blinding of outcome assessment (detection bias)	High risk	As above
Incomplete outcome data (attrition bias)	Low risk	Less than 1.5% of participants were lost to follow up, and primary outcome data was unavailable for a further 1.6%
Selective reporting (reporting bias)	Low risk	Outcomes reported as per protocol (NCT01462422)

Shady 2017

Domain	Review authors judgement	Support for judgement
Random sequence generation (selection bias)	Low risk	A computer-generated randomisation table was used
Allocation concealment (selection bias)	Low risk	Allocation data was placed in "serially numbered closed opaque envelopes"
Blinding of participants and personnel (performance bias)	High risk	This was an open label trial, so blinding would not be possible.
Blinding of outcome assessment (detection bias)	High risk	As above
Incomplete outcome data (attrition bias)	Low risk	All data from randomised participants were analysed and reported
Selective reporting (reporting bias)	Unclear risk	There is an unclear definition of postpartum haemorrhage.

Tita 2012

Domain	Review authors judgement	Support for judgement
Random sequence generation (selection bias)	Low risk	"Women were randomized to one of the three study arms according to a confidential computer-generated block randomization algorithm. The algorithm randomly allocated three women to each study dose in blocks of nine, thus ensuring equal allocation among the study arms"
Allocation concealment (selection bias)	Low risk	Preparation of the oxytocin was done prior to the randomisation process and by research members who had "no role in patient enrolment or outcome ascertainment"
Blinding of participants and personnel (performance bias)	Low risk	Participants and personnel (who had patient contact) were blinding to the concentration of oxytocin in the numbered bags
Blinding of outcome assessment (detection bias)	Low risk	"Only the investigational pharmacist and one statistician, who had no role in patient enrolment or outcome ascertainment"
Incomplete outcome data (attrition bias)	Low risk	Data was analysed for all participants who had been randomised
Selective reporting (reporting bias)	Low risk	All outcomes reported had been previously described in the published registration (NCT00790062)
