# Supplementary material for: Postpartum haemorrhage (PPH) rates in randomized trials of PPH prophylactic interventions and the effect of underlying participant PPH risk: a meta-analysis
Source: BMC Pregnancy Childbirth. 2020 Feb 13;20:107. doi: 10.1186/s12884-020-2719-3 (PMC7020586; doi:10.1186/s12884-020-2719-3)
Supplement: Supplementary file 2 — Additional file 2. Proportion meta-analysis box plots for the individual grades for minor postpartum haemorrhage. [file 12884_2020_2719_MOESM2_ESM.pdf]

Additional File 2 – Proportion meta-analysis box plots for the individual grades for minor postpartum haemorrhage

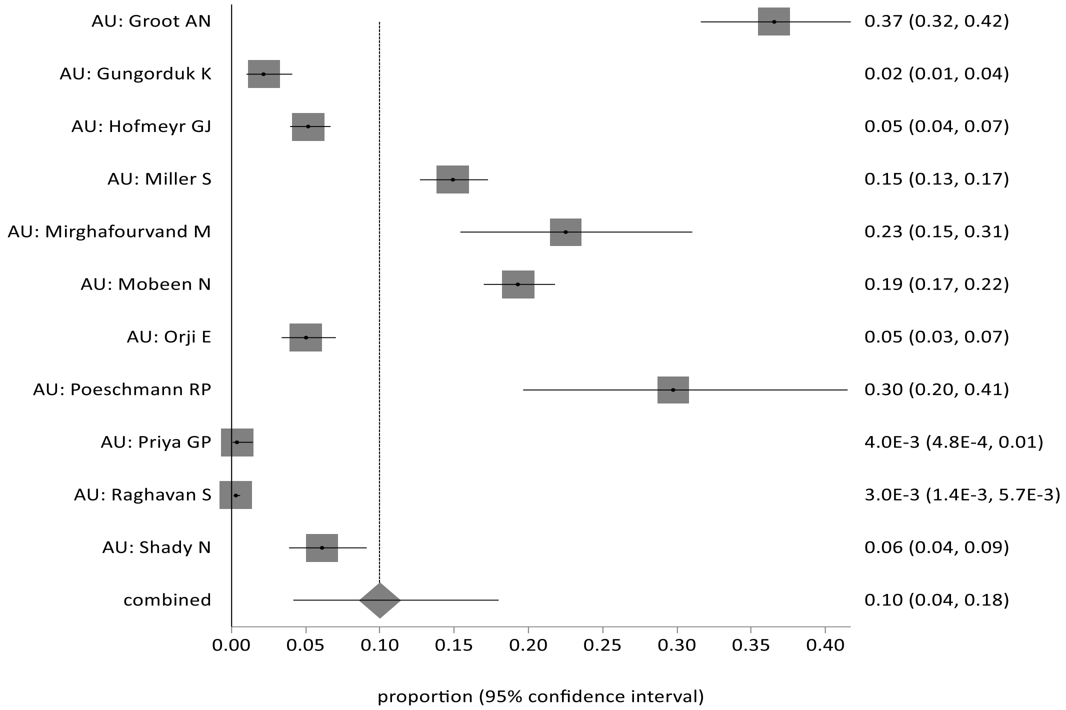

Figure 1. Trials included in Grade 1 (low risk antenatally and 'normal' vaginal births only), with reported trial rate for minor PPH. Pooled proportion of 0.10 (95% CI = 0.042 to 0.18).

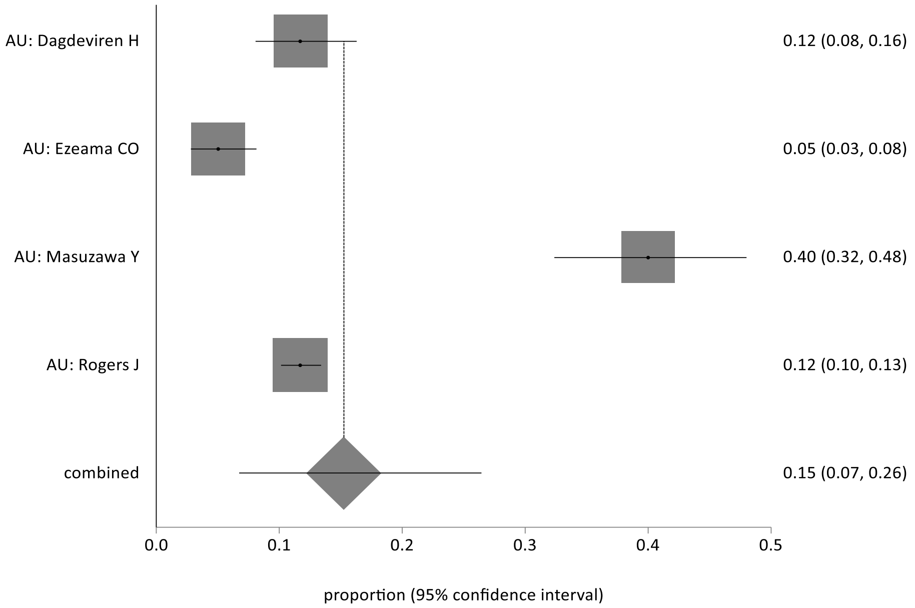

Figure 2. Trials included in Grade 2 (low risk antenatally and <10% operative births), with reported trial rate for minor PPH. Pooled proportion of 0.15 (95% CI = 0.067 to 0.26).

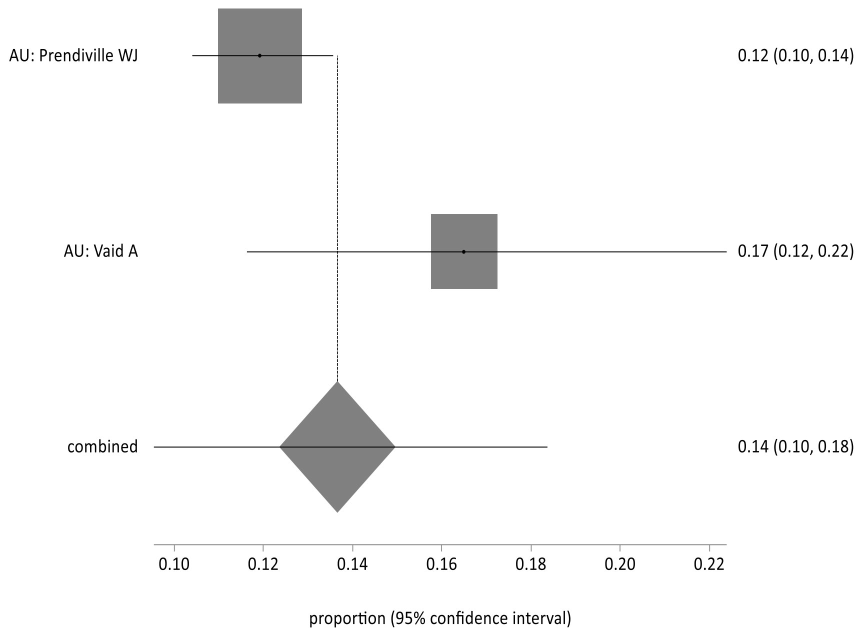

Figure 3. Trials included in Grade 3 (low risk antenatally and ≥10% operative births), with reported trial rate for minor PPH. Pooled proportion of 0.14 (95% CI = 0.095 to 0.18).

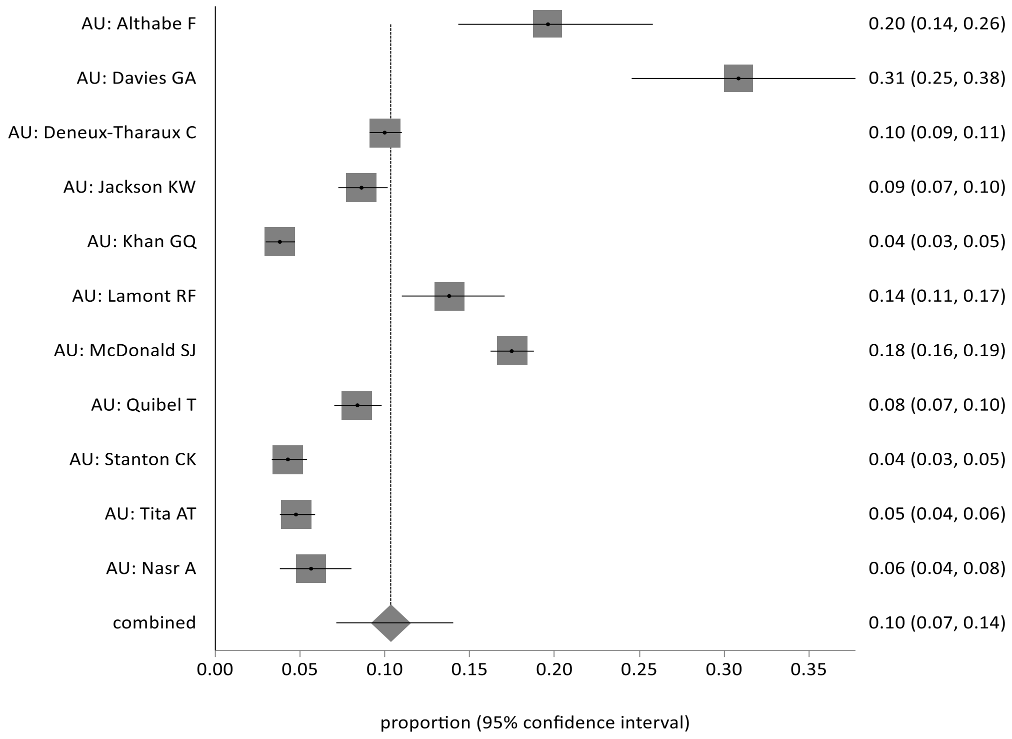

Figure 4. Trials included in Grade 4 (unselected antenatally and all births), with reported trial rate for minor PPH. Pooled proportion of 0.10 (95% CI = 0.072 to 0.14).
