# Supplementary material for: Postpartum haemorrhage (PPH) rates in randomized trials of PPH prophylactic interventions and the effect of underlying participant PPH risk: a meta-analysis
Source: BMC Pregnancy Childbirth. 2020 Feb 13;20:107. doi: 10.1186/s12884-020-2719-3 (PMC7020586; doi:10.1186/s12884-020-2719-3)
Supplement: Supplementary file 3 — Additional file 3. Proportion meta-analysis box plots for the individual grades for major postpartum haemorrhage. [file 12884_2020_2719_MOESM3_ESM.pdf]

Additional File 3 - Proportion meta-analysis box plots for the individual grades for major postpartum haemorrhage

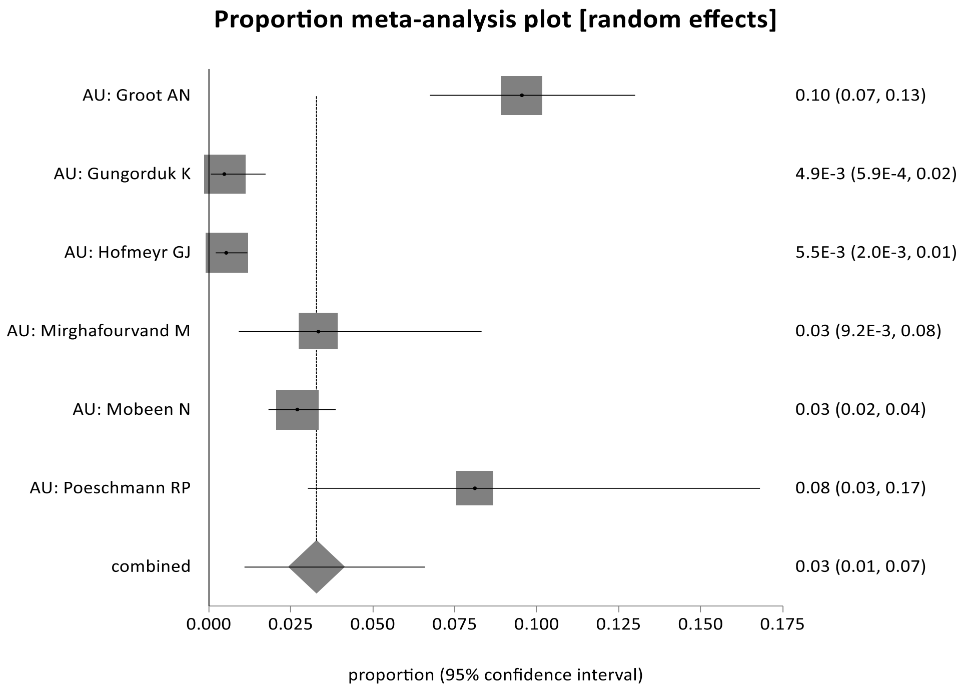

Figure 1. Trials included in Grade 1 (low risk antenatally and 'normal' vaginal births only), with reported trial rate for major PPH. Pooled proportion of 0.033 (95% CI = 0.011 to 0.066).

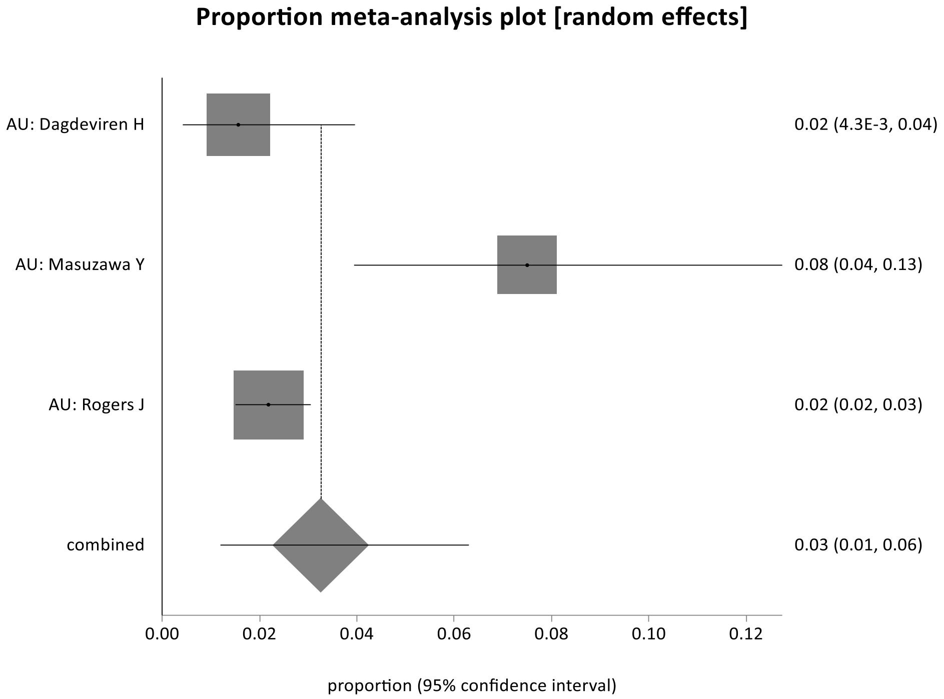

Figure 2. Trials included in Grade 2 (low risk antenatally and <10% operative births), with reported trial rate for major PPH. Pooled proportion of 0.033 (95% CI = 0.012 to 0.063).

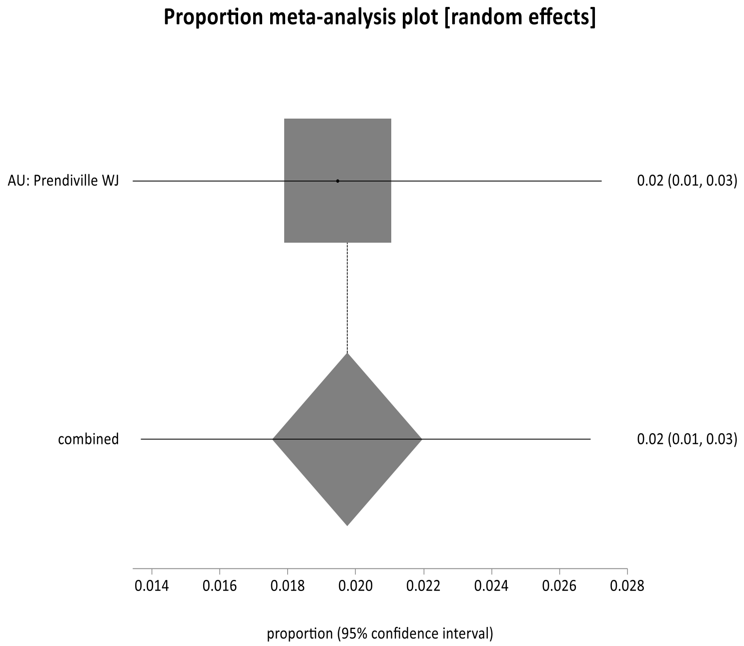

Figure 3. Trials included in Grade 3 (low risk antenatally and ≥10% operative births), with reported trial rate for major PPH. Pooled proportion of 0.020 (95% CI = 0.014 to 0.027).

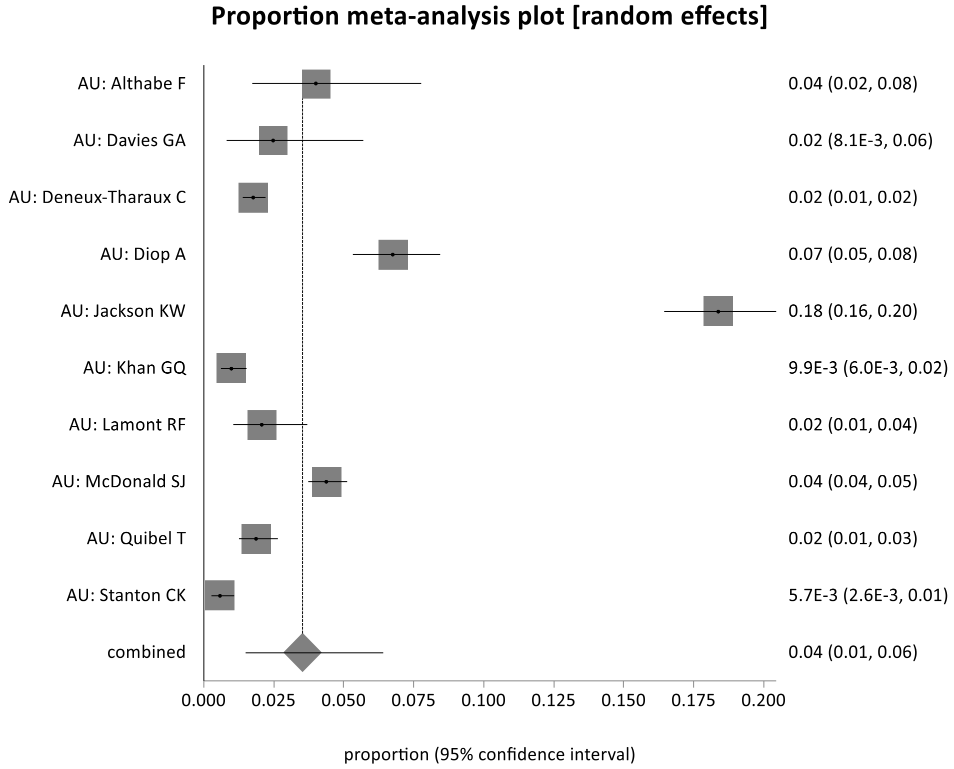

Figure 4. Trials included in Grade 4 (unselected antenatally and all births), with reported trial rate for major PPH. Pooled proportion of 0.035 (95% CI = 0.015 to 0.064).

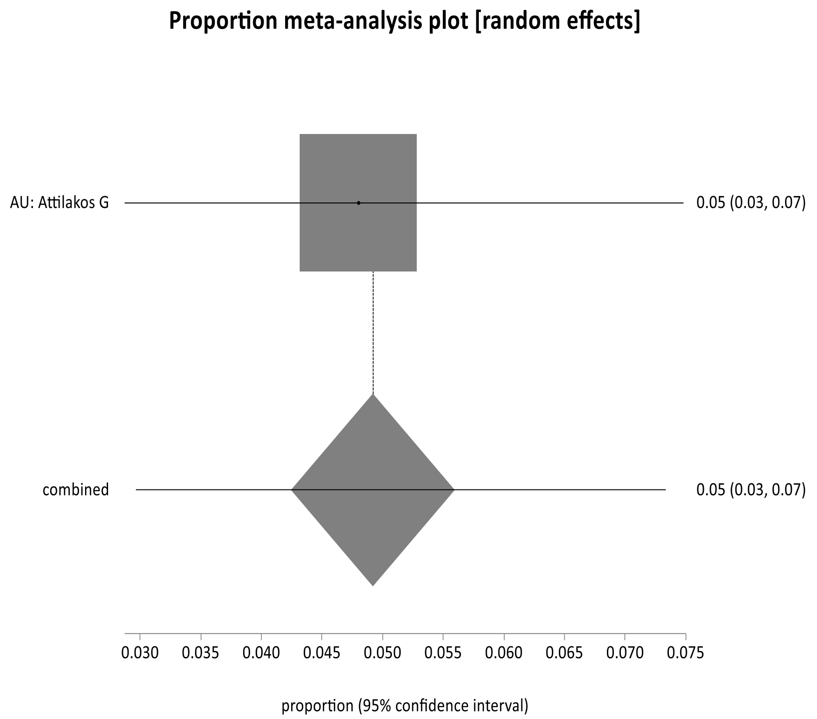

Figure 5. Trials included in Grade 5 (unselected antenatally and operative births only), with reported trial rate for major PPH. Pooled proportion of 0.049 (95% CI = 0.030 to 0.073).
