# Supplementary material for: Postpartum haemorrhage (PPH) rates in randomized trials of PPH prophylactic interventions and the effect of underlying participant PPH risk: a meta-analysis
Source: BMC Pregnancy Childbirth. 2020 Feb 13;20:107. doi: 10.1186/s12884-020-2719-3 (PMC7020586; doi:10.1186/s12884-020-2719-3)
Supplement: Supplementary file 4 — Additional file 4. Table of trials included in “A systematic review of postpartum haemorrhage (PPH) rates in randomized trials of PPH prophylactic interventions to examine the effect of underlying participant PPH risk”. [file 12884_2020_2719_MOESM4_ESM.pdf]

Additional File 4 - Table of trials included in “A systematic review of postpartum haemorrhage (PPH) rates in randomized trials of PPH prophylactic interventions to examine the effect of underlying participant PPH risk”.

**Authors**	**Trial**	**DOI**
Groot AN *et al* 1996[1]	A placebo-controlled trial of oral ergometrine to reduce postpartum hemorrhage	doi.org/10.3109/00016349609033355
Gungorduk K *et al* 2010[2]	Using intraumbilical vein injection of oxytocin in routine practice with active management of the third stage of labor: a randomized controlled trial	doi.org/10.1097/AOG.0b013e3181edac6b
Hofmeyr G *et al* 2011[3]	Administration of 400μg of misoprostol to augment routine active management of the third stage of labor	doi.org/10.1016/j.ijgo.2010.08.019
Miller S *et al* 2009[4]	Randomized double masked trial of Zhi Byed 11, a Tibetan traditional medicine, versus misoprostol to prevent postpartum hemorrhage in Lhasa, Tibet	doi.org/10.1016/j.jmwh.2008.09.010
Mirghafourvand M *et al* 2015[5]	The effect of prophylactic intravenous tranexamic acid on blood loss after vaginal delivery in women at low risk of postpartum haemorrhage: a double-blind randomised controlled trial	doi.org/10.1111/ajo.12262
Mobeen N *et al* 2011[6]	Administration of misoprostol by trained traditional birth attendants to prevent postpartum haemorrhage in homebirths in Pakistan: a randomised placebo-controlled trial	doi.org/10.1111/j.1471-0528.2010.02807.x
Orji E *et al* 2008[7]	A randomized comparative study of prophylactic oxytocin versus ergometrine in the third stage of labor	doi.org/10.1016/j.ijgo.2007.11.009
Poeschmann RP *et al* 1991[8]	A randomized comparison of oxytocin, sulprostone and placebo in the management of the third stage of labour	doi.org/10.1111/j.1471-0528.1991.tb10364.x
Priya GP *et al* 2015[9]	A randomized controlled trial of sublingual misoprostol and intramuscular oxytocin for prevention of postpartum hemorrhage	doi.org/10.1007/s00404-015-3763-5
Raghavan S *et al* 2016[10]	Misoprostol for primary versus secondary prevention of postpartum haemorrhage: a cluster-randomised non-inferiority community trial	doi.org/10.1111/1471-0528.13540
Shady NW *et al* 2017[11]	The effect of prophylactic oral tranexamic acid plus buccal misoprostol on blood loss after vaginal delivery: a randomized controlled trial	doi.org/10.1080/14767058.2017.1418316
Dagdeviren H *et al* 2016[12]	Intramuscular versus intravenous prophylactic oxytocin for postpartum hemorrhage after vaginal delivery: a randomized controlled study	doi.org/10.1007/s00404-016-4060-7

Authors	Trial	DOI
Ezeama CO *et al* 2014[13]	A comparison of prophylactic intramuscular ergometrine and oxytocin for women in the third stage of labor	doi.org/10.1016/j.ijgo.2013.07.020
Masuzawa Y *et al* 2017[14]	Cooling the lower abdomen to reduce postpartum blood loss: a randomized controlled trial	doi.org/10.1371/journal.pone.0186365
Rogers J *et al* 1998[15]	Active versus expectant management of third stage of labour: the Hinchingbrooke randomised controlled trial	doi.org/10.1016/S0140-6736(97)09409-9
Prendiville WJ *et al* 1988[16]	The Bristol third stage trial: active versus physiological management of third stage of labour	doi.org/10.1136/bmj.297.6659.1295
Vaid A *et al* 2009[17]	A randomized controlled trial of prophylactic sublingual misoprostol versus intramuscular methyl-ergometrine versus intramuscular 15-methyl PGF2alpha in active management of third stage of labor	doi.org/10.1007/s00404-009-1019-y
Althabe F *et al* 2009[18]	A pilot randomized controlled trial of controlled cord traction to reduce postpartum blood loss	doi.org/10.1016/j.ijgo.2009.05.021
Davies GA *et al* 2005[19]	Maternal hemodynamics after oxytocin bolus compared with infusion in the third stage of labor: a randomized controlled trial	doi.org/10.1097/01.AOG.0000162351.93494.6d
Deneux-Tharaux C *et al* 2013[20]	Effect of routine controlled cord traction as part of the active management of the third stage of labour on postpartum haemorrhage: multicentre randomised controlled trial (TRACOR)	doi.org/10.1136/bmj.f1541
Diop A *et al* 2016[21]	Oxytocin via Uniject (a prefilled single-use injection) versus oral misoprostol for prevention of postpartum haemorrhage at the community level: a cluster-randomised controlled trial	doi.org/10.1016/S2214-109X
Jackson KW *et al* 2001[22]	A randomized controlled trial comparing oxytocin administration before and after placental delivery in the prevention of postpartum hemorrhage	doi.org/10.1067/mob.2001.117363
Khan GQ *et al* 1995[23]	Abu Dhabi third stage trial: oxytocin versus Syntometrine in the active management of the third stage of labour	doi.org/10.1016/0028-2243(95)80014-J
Lamont RF *et al* 2001[24]	A prospective randomised trial to compare the efficacy and safety of hemabate and syntometrine for the prevention of primary postpartum haemorrhage	doi.org/10.1016/S0090-6980(01)00154-X
McDonald SJ *et al* 1993[25]	Randomised controlled trial of oxytocin alone versus oxytocin and ergometrine in active management of third stage of labour	doi.org/10.1136/bmj.307.6913.1167
Nasr A *et al* 2009[26]	Rectal misoprostol versus intravenous oxytocin for prevention of postpartum hemorrhage	doi.org/10.1016/j.ijgo.2009.01.018

Authors	Trial	DOI
Quibel T *et al* 2016[27]	Active Management of the Third Stage of Labor With a Combination of Oxytocin and Misoprostol to Prevent Postpartum Hemorrhage: a Randomized Controlled Trial	doi.org/10.1097/AOG.0000000000001626
Stanton CK *et al* 2013[28]	Effect on postpartum hemorrhage of prophylactic oxytocin (10 IU) by injection by community health officers in Ghana: a community-based, cluster-randomized trial	doi.org/10.1371/journal.pmed.1001524
Tita AT *et al* 2012[29]	Higher-dose oxytocin and hemorrhage after vaginal delivery: a randomized controlled trial	doi.org/10.1097/AOG.0b013e318242da74
Attilakos G *et al* 2010[30]	Carbetocin versus oxytocin for the prevention of postpartum haemorrhage following caesarean section: the results of a double-blind randomised trial	doi.org/10.1111/j.1471-0528.2010.02585.x

References

1. Groot ANJAD, Roosmalen JV, Dongen PWJV, Borm GF. A placebo-controlled trial of oral ergometrine to reduce postpartum haemorrhage. *Acta Obstetrica et Gynecologica Scandanavica* 1996;75:464-468.
2. Güngördük K, Asicioglu O, Besimoglu B, Güngördük OC, Yildirm G, Ark C, et al. Using Intraumbilical Vein Injection of Oxytocin in Routine Practice With Active Management of the Third Stage of Labor: A Randomized Controlled Trial. *Obstetrics & Gynecology* 2010;116(3):619-24.
3. Hofmeyr GJ, Fawole B, Mugerwa K, Godi NP, Blignaut Q, Mangesi L, et al. Administration of 400 µg of misoprostol to augment routine active management of the third stage of labor. *International Journal of Gynecology & Obstetrics* 2011;112(2):98-102.
4. Miller S, Tudor C, Thorsten V, Nyima, Kalyang, Sonam, et al. Randomized double masked trial of Zhi Byed 11, a Tibetan traditional medicine, versus misoprostol to prevent postpartum hemorrhage in Lhasa, Tibet. *Journal of Midwifery & Womens Health* 2009;54(2):133-41.e1.
5. Mirghafourvand M, Mohammad-Alizadeh S, Abbasalizadeh F, Shirdel M. The effect of prophylactic intravenous tranexamic acid on blood loss after vaginal delivery in women at low risk of postpartum haemorrhage: a double-blind randomised controlled trial. *Australian and New Zealand Journal of Obstetrics and Gynaecology* 2015;55(1):53-8.
6. Mobeen N, Durocher J, Zuberi N, Jahan N, Blum J, Wasim S, et al. Administration of misoprostol by trained traditional birth attendants to prevent postpartum haemorrhage in homebirths in Pakistan: a randomised placebo-controlled trial. *BJOG: An International Journal of Obstetrics & Gynaecology* 2011;118(3):353-61.
7. Orji E, Agwu F, Loto O, Olaleye O. A randomized comparative study of prophylactic oxytocin versus ergometrine in the third stage of labor. *International Journal of Gynaecology & Obstetrics* 2008;101(2):129-32.
8. Poeschmann RP, Doesburg WH, Eskes TKAB. A randomized comparison of oxytocin, sulprostone and placebo in the management of the third stage of labour. *BJOG: An International Journal of Obstetrics & Gynaecology* 1991;98(6):528-30.
9. Priya GP, Veena P, Chaturvedula L, Subitha L. A randomized controlled trial of sublingual misoprostol and intramuscular oxytocin for prevention of postpartum hemorrhage. *Archives of Gynecology & Obstetrics* 2015;292(6):1231-7.

10. Raghavan S, Geller S, Miller S, Goudar S, Anger H, Yadavannavar M, et al. Misoprostol for primary versus secondary prevention of postpartum haemorrhage: a cluster-randomised non-inferiority community trial. *BJOG: An International Journal of Obstetrics & Gynaecology* 2016;123(1):120-7.
11. Shady NW, Sallam HF, Elsayed AH, Abdelkader AM, Ali SS, Alanwar A, et al. The effect of prophylactic oral tranexamic acid plus buccal misoprostol on blood loss after vaginal delivery: a randomized controlled trial. *Journal of Maternal-Fetal & Neonatal Medicine* 2017:1-7.
12. Dagdeviren H, Cengiz H, Heydarova U, Caypinar SS, Kanawati A, Guven E, et al. Intramuscular versus intravenous prophylactic oxytocin for postpartum hemorrhage after vaginal delivery: a randomized controlled study. *Archives of Gynecology & Obstetrics* 2016;294(5):911-6.
13. Ezeama CO, Eleje GU, Ezeama NN, Igwegbe AO, Ikechebelu JI, Ugboaja JO, et al. A comparison of prophylactic intramuscular ergometrine and oxytocin for women in the third stage of labor. *International Journal of Gynaecology & Obstetrics* 2014;124(1):67-71.
14. Masuzawa Y, Kataoka Y, Nakamura S, Yaju Y. Cooling the lower abdomen to reduce postpartum blood loss: A randomized controlled trial. *PLOS ONE* 2017;12(10):e0186365.
15. Rogers J, Wood J, McCandlish R, Ayers S, Truesdale A, Elbourne D. Active versus expectant management of third stage of labour: the Hinchingbrooke randomised controlled trial. *Lancet* 1998;351(9104):693-9.
16. Prendiville WJ, Harding JE, Elbourne DR, Stirrat GM. The Bristol third stage trial: active versus physiological management of third stage of labour. *British Medical Journal* 1988;297(6659):1295.
17. Vaid A, Dadhwal V, Mittal S, Deka D, Misra R, Sharma JB, et al. A randomized controlled trial of prophylactic sublingual misoprostol versus intramuscular methyl-ergometrine versus intramuscular 15-methyl PGF2alpha in active management of third stage of labor. *Archives of Gynecology & Obstetrics* 2009;280(6):893-7.
18. Althabe F, Alemán A, Tomasso G, Gibbons L, Vitureira G, Belizán JM, et al. A pilot randomized controlled trial of controlled cord traction to reduce postpartum blood loss. *International journal of gynaecology and obstetrics: the official organ of the International Federation of Gynaecology and Obstetrics* 2009;107(1):4-7.
19. Davies GA, Tessier JL, Woodman MC, Lipson A, Hahn PM. Maternal hemodynamics after oxytocin bolus compared with infusion in the third stage of labor: a randomized controlled trial. *Obstetrics & Gynecology* 2005;105(2):294-9.
20. Deneux-Tharaux C, Sentilhes L, Maillard F, Closset E, Vardon D, Lepercq J, et al. Effect of routine controlled cord traction as part of the active management of the third stage of labour on postpartum haemorrhage: multicentre randomised controlled trial (TRACOR). *BMJ* 2013;346:f1541.
21. Diop A, Daff B, Sow M, Blum J, Diagne M, Sloan NL, et al. Oxytocin via Uniject (a prefilled single-use injection) versus oral misoprostol for prevention of postpartum haemorrhage at the community level: a cluster-randomised controlled trial. *The Lancet Global Health* 2016;4(1):e37-e44.
22. Jackson KW, Jr., Allbert JR, Schemmer GK, Elliot M, Humphrey A, Taylor J. A randomized controlled trial comparing oxytocin administration before and after placental delivery in the prevention of postpartum hemorrhage. *American Journal of Obstetrics & Gynecology* 2001;185(4):873-7.
23. Khan GQ, John IS, Chan T, Wani S, Hughes AO, Stirrat GM. Abu Dhabi third stage trial: oxytocin versus Syntometrine in the active management of the third stage of labour. *European Journal of Obstetrics & Gynecology and Reproductive Biology* 1995;58(2):147-51.
24. Lamont RF, Morgan DJ, Logue M, Gordon H. A prospective randomised trial to compare the efficacy and safety of hemabate and syntometrine for the prevention of primary postpartum haemorrhage. *Prostaglandins & Other Lipid Mediators* 2001;66(3):203-10.

25. McDonald SJ, Prendiville WJ, Blair E. Randomised controlled trial of oxytocin alone versus oxytocin and ergometrine in active management of third stage of labour. *BMJ* 1993;307(6913):1167-71.
26. Nasr A, Shahin AY, Elsamman AM, Zakherah MS, Shaaban OM. Rectal misoprostol versus intravenous oxytocin for prevention of postpartum hemorrhage. *Internation Journal of Gynaecology & Obstetrics* 2009;105(3):244-7.
27. Quibel T, Ghout I, Goffinet F, Salomon LJ, Fort J, Javoise S, et al. Active Management of the Third Stage of Labor With a Combination of Oxytocin and Misoprostol to Prevent Postpartum Hemorrhage: A Randomized Controlled Trial. *Obstetrics & Gynecology* 2016;128(4):805-11.
28. Stanton CK, Newton S, Mullany LC, Cofie P, Tawiah Agyemang C, Adiibokah E, et al. Effect on Postpartum Hemorrhage of Prophylactic Oxytocin (10 IU) by Injection by Community Health Officers in Ghana: A Community-Based, Cluster-Randomized Trial. *PLOS Medicine* 2013;10(10):e1001524.
29. Tita AT, Szychowski JM, Rouse DJ, Bean CM, Chapman V, Nothern A, et al. Higher-dose oxytocin and hemorrhage after vaginal delivery: a randomized controlled trial. *Obstetrics & Gynecology* 2012;119(2 Pt 1):293-300.
30. Attilakos G, Psaroudakis D, Ash J, Buchanan R, Winter C, Donald F, et al. Carbetocin versus oxytocin for the prevention of postpartum haemorrhage following caesarean section: the results of a double-blind randomised trial. *BJOG* 2010;117(8):929-36.
